# Photophysical properties and fluorescence lifetime imaging of exfoliated near-infrared fluorescent silicate nanosheets†

**DOI:** 10.1039/d1na00238d

**Published:** 2021-06-24

**Authors:** Gabriele Selvaggio, Milan Weitzel, Nazar Oleksiievets, Tabea A. Oswald, Robert Nißler, Ingo Mey, Volker Karius, Jörg Enderlein, Roman Tsukanov, Sebastian Kruss

**Affiliations:** Physical Chemistry II, Bochum University Bochum 44801 Germany Sebastian.Kruss@rub.de; Institute of Physical Chemistry, University of Göttingen Göttingen 37077 Germany; Third Institute of Physics, University of Göttingen Göttingen 37077 Germany; Institute of Organic and Biomolecular Chemistry, University of Göttingen Göttingen 37077 Germany; Department of Sedimentology and Environmental Geology, Geoscience Center, University of Göttingen Göttingen 37077 Germany; Cluster of Excellence “Multiscale Bioimaging: from Molecular Machines to Networks of Excitable Cells” (MBExC), University of Göttingen Germany; Fraunhofer Institute for Microelectronic Circuits and Systems Duisburg 47057 Germany

## Abstract

The layered silicates Egyptian Blue (CaCuSi_4_O_10_, EB), Han Blue (BaCuSi_4_O_10_, HB) and Han Purple (BaCuSi_2_O_6_, HP) emit as bulk materials bright and stable fluorescence in the near-infrared (NIR), which is of high interest for (bio)photonics due to minimal scattering, absorption and phototoxicity in this spectral range. So far the optical properties of nanosheets (NS) of these silicates are poorly understood. Here, we exfoliate them into monodisperse nanosheets, report their physicochemical properties and use them for (bio)photonics. The approach uses ball milling followed by tip sonication and centrifugation steps to exfoliate the silicates into NS with lateral size and thickness down to ≈ 16–27 nm and 1–4 nm, respectively. They emit at ≈ 927 nm (EB-NS), 953 nm (HB-NS) and 924 nm (HP-NS), and single NS can be imaged in the NIR. The fluorescence lifetimes decrease from ≈ 30–100 μs (bulk) to 17 μs (EB-NS), 8 μs (HB-NS) and 7 μs (HP-NS), thus enabling lifetime-encoded multicolor imaging both on the microscopic and the macroscopic scale. Finally, remote imaging through tissue phantoms reveals the potential for bioimaging. In summary, we report a procedure to gain monodisperse NIR fluorescent silicate nanosheets, determine their size-dependent photophysical properties and showcase the potential for NIR photonics.

## Abstract 2

Egyptian Blue (CaCuSi_4_O_10_, EB), Han Blue (BaCuSi_4_O_10_, HB) and Han Purple (BaCuSi_2_O_6_, HP) are exfoliated into 2D nanosheets (NS). Near-infrared (NIR) fluorescence properties are reported and (lifetime) imaging applications are demonstrated.

## Graphical abstract

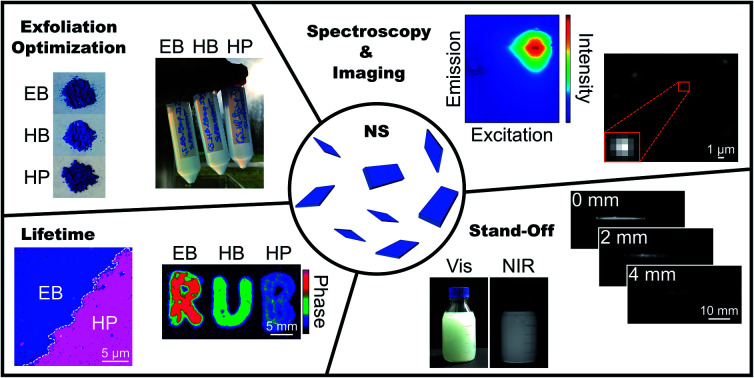

## Introduction

Two-dimensional (2D) nanomaterials have attracted considerable interest in light of their exceptional photophysical properties and their potential for multiple applications.^1–4^ Following graphene, other 2D materials such as transition metal dichalcogenides (TMDs) have been explored.^4–6^ Single layers of 2D TMDs possess a bandgap and show versatile chemistry.^1,4,7–9^ Because of the very high surface area displayed by these nanostructures, TMD nanosheets (NS) have been employed in catalysis, energy storage, sensing, and electronics.^1,4,5,7,9–14^

Bottom-up synthetic techniques for 2D materials (*e.g.* chemical vapor deposition (CVD), epitaxial growth, or wet chemical methods^7,8^) enable fabrication of large-area, ultrathin and uniform layers but at high expenses.^9,14^ An alternative is top-down exfoliation (or delamination), which presents several advantages. Liquid-phase exfoliation (LPE) promises scalability, subsequent chemical modification as well as simple deposition on diverse surfaces.^1,9,14^ With ultrasonication, NS sizes of few hundred nanometers are achievable.^9^ Scalable high shear processing techniques such as wet ball milling are also known to yield monodisperse samples and are commonly employed tools for all kind of layered materials *e.g.* sheet silicates.^14–16^

A novel class of 2D materials are (phyllo)silicates such as Egyptian Blue (CaCuSi_4_O_10_, EB), which is as (exfoliated) nanosheet a promising fluorophore for photonics.^17,18^ This calcium copper tetrasilicate is regarded as the most ancient artificial pigment made by mankind and dates back to Ancient Egypt (≈ 2500 BC), where it was employed in artwork.^19^ EB's tetragonal crystal structure (space group *P*4/*ncc*) consists of parallel layers of silicate tetrahedra weakly held together by the presence of interlayer calcium ions in an eight-fold coordination geometry.^20^ The copper ions are placed in a square planar coordination geometry and are most likely the reason for the photophysical properties of this material. It was reported that pristine EB emits in the near-infrared (NIR) region at ≈ 910–930 nm, with a broad excitation spectrum at green-red wavelengths.^21–23^ EB fluorescence is characterized by a very high quantum yield (≈ 11%^23^) compared to typical NIR fluorescent dyes^23,24^ and a long lifetime (*τ* ≈ 100–150 μs).^22,23,25^ Theoretical investigations into electronic and magnetic properties of bulk and monolayer EB have suggested that EB could turn into a direct band-gap semiconductor if its thickness is reduced down to the monolayer regime (≈ 1 nm).^19,25,26^ The exfoliation of EB into μm-sized nanosheets (EB-NS) is possible by stirring in hot water over several days.^1,6,27–30^

There are also homologues of EB such as Han Blue (BaCuSi_4_O_10_, HB) and Han Purple (BaCuSi_2_O_6_, HP). While the former presents a crystal structure identical to EB's (except for the substitution of Ca^2+^ with Ba^2+^), the latter is less rich in silica and presents a chemically labile Cu–Cu bond, which is likely the explanation behind a weaker stability of this compound towards weak acids.^26,31,32^ HB and HP are also fluorescent in the NIR, and this can be attributed to the Cu^2+^ ions and rationalized by ligand-field theory.^22^ The interesting photophysical properties of EB, HB and HP have so far been mostly exploited to classify and assess the origin of ancient artwork.^23,33,34^ Nevertheless, applications have been proposed as ink, fingerprint dusting powder, scaffold for selective enrichment of phosphopeptides or luminescent solar concentrators.^27,35–39^ Recently, it was shown that EB nanosheets (EB-NS) keep their NIR fluorescence properties and can serve as NIR fluorophore.^17^

In general, fluorescence methods using the NIR (800–1700 nm) benefit from reduced scattering, absorption and photodamage, leading to better image contrast and, in biological applications, to better tissue penetration.^40,41^ Moreover, phototoxicity is also lower compared to other wavelengths.^24,42,43^ Unfortunately, there exist only few NIR fluorophores, and most are photolabile, possess low quantum yields, or have biocompatibility issues. In contrast to organic fluorophores (*e.g.* indocyanine green (ICG)), nanomaterials such as InAs quantum dots, lanthanide-doped nanoparticles, or semiconducting single-walled carbon nanotubes (SWCNTs) offer an enhanced photostability and a handle for chemical functionalization.^44–49^ Especially for SWCNTs, chemical concepts have been developed to conjugate a broad range of biomolecules, which is required for molecular recognition.^18,50–54^ Nevertheless, there is a large demand for novel NIR fluorophores with better or complementary properties.

Here, we exfoliate EB, HB and HP into nanosheets (EB-NS, HB-NS and HP-NS) *via* a mixed approach of planetary ball milling (PB) and tip sonication (TS) in water. This route enables us access to NS of defined size distribution, of which we report properties including fluorescence spectra and lifetimes. Finally, we show the potential of these materials for biophotonic applications such as lifetime-encoded imaging or deep-tissue imaging.

## Results and discussion

### Exfoliation process and size distribution

**Fig. 1:**
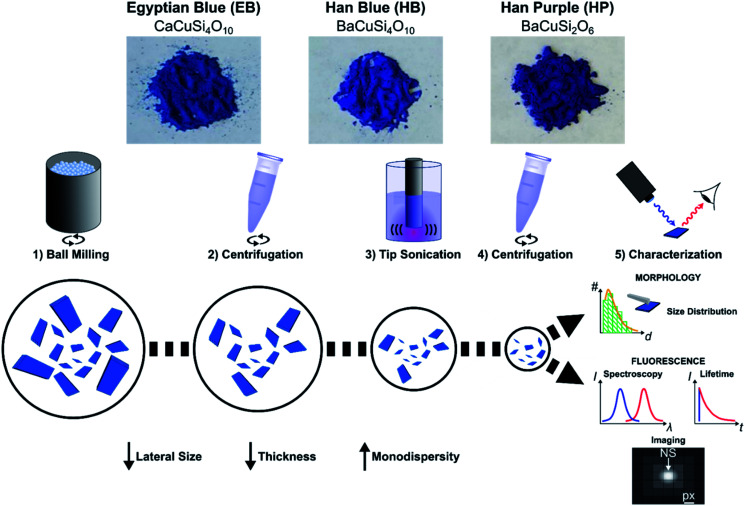
Exfoliation of layered silicates into nanosheets. Bulk Egyptian Blue (CaCuSi_4_O_10_, EB), Han Blue (BaCuSi_4_O_10_, HB) and Han Purple (BaCuSi_2_O_6_, HP) are exfoliated into nanosheets (EB-NS, HB-NS and HP-NS). The bulk crystals are first reduced in size by a planetary ball mill (PB) in water. *Via* centrifugation larger particles are removed, then the supernatant is tip sonicated (6 h). An additional centrifugation step is performed to further increase monodispersity. These NS are colloidally stable and their properties such as the near-infrared (NIR) fluorescence are characterized and used for (bio)photonics.

To gain access to nanosheets of defined height and lateral size, we developed a multi-step exfoliation procedure, which was optimized for EB, HB and HP (Fig. 1).

**Fig. 2:**
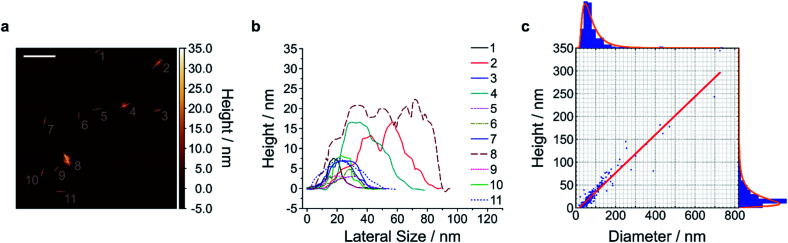
Size and height of exfoliated silicate nanosheets. Exfoliated nanosheets were spin-coated on mica and imaged with atomic force microscopy (AFM) in intermittent contact mode. A representative image (a) and the respective height traces (b) and histograms with log-normal fits (c) of EB-NS are shown. The results indicate that lateral size scales linearly with height (linear fit = red line in (c)). The diameter/height values corresponding to the log-normal distributions' maxima are 47.0 nm/9.0 nm, with *R*^2^ values of the linear fit of 0.924. *n* = 245 measured NS. The exfoliation of HB-NS and HP-NS yielded similar outcomes (Fig. S12†). Scale bar = 200 nm.

The first step of this protocol uses planetary ball milling in water, which is frequently employed to crush minerals down to few μm-sized crystallites.^16,55^ In a second step, the obtained slurry was centrifuged to remove larger particles. The supernatant (pH ≈ 10–11), which already showed an increased colloidal stability in water (Fig. S1 and S2†), contained μm-sized objects as measured by laser diffraction (Fig. S3–S6†). Next, the milled supernatant was tip sonicated to further decrease lateral size and height. Finally, the sample was centrifuged to improve monodispersity and to mainly obtain the smallest NS. The final concentration of the EB-NS, HB-NS and HP-NS was ≈ 0.5 g L^−1^ and, compared to the bulk counterparts, all samples were colloidally stable up to several days (pH ≈ 8–9, Fig. S7†). Furthermore, we observed that the colloidal stability of these NS was not significantly affected by acidic conditions and ionic environments such as physiological buffers (Fig. S8–S11†). Atomic force microscopy (AFM) and scanning electron microscopy (SEM) were used to assess size distribution and morphology of the NS. The exfoliated EB-NS, HB-NS and HP-NS show lateral sizes of few tenths of nm and heights ranging from monolayers to multilayers (Fig. 2 and S12a–f†). The data furthermore showed (Fig. S12g–i†) that lateral size increases linearly with thickness. As expected from the fragmentation of crystal structures like silicates undergoing sonication and/or milling, a log-normal function can best describe the overall trend of both lateral size and thickness of EB-NS, HB-NS and HP-NS.^55^ Scanning electron microscopy (SEM) revealed additional insights into particle morphology (Fig. S13 and S14a–c†). This was also evaluated *via* scanning transmission electron microscopy (STEM, Fig. S14†). As indicated by AFM, SEM and STEM (Fig. S14d–f†), the exfoliation yields small NS with varying morphology, which could be attributed to the mechanical stress applied during milling and tip sonication. Nevertheless, we also observed NS with very regular geometry (Fig. S14d–f, S15 and S16†).

### NIR spectroscopy and imaging

During transition from bulk to the nanoscale material, one could imagine that the NIR fluorescence might be damaged/destroyed. We therefore studied the NIR fluorescence of the NS.

**Fig. 3:**
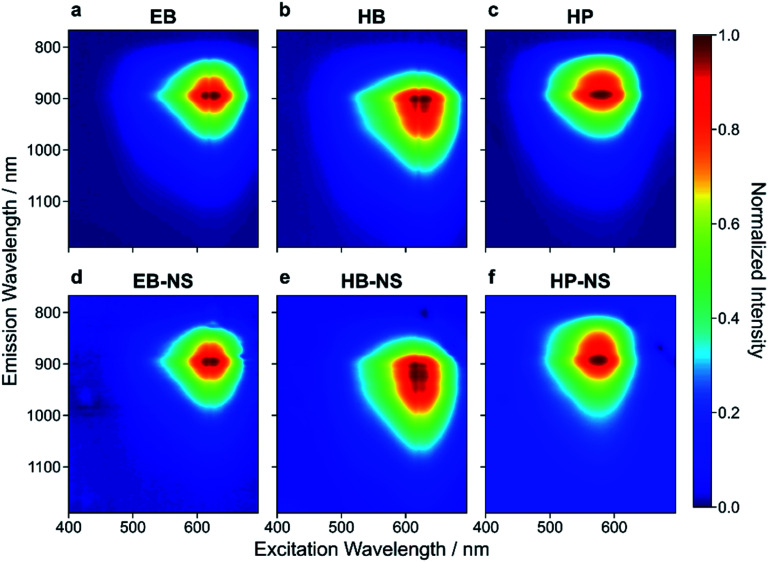
Excitation–emission spectra of exfoliated silicate nanosheets. Normalized 2D excitation–emission spectra of bulk (a–c) and exfoliated (d–f) EB, HB and HP. The exfoliation procedure and decrease of size/layer number has nearly no influence on the emission spectra. Absorption is maximal at ≈ 630 nm (EB-NS), ≈ 620 nm (HB-NS) and ≈ 580 nm (HP-NS). These 2D spectra as well as 1D spectra (*λ*_exc_ = 561 nm, Fig. S18†) confirm emission maxima at ≈ 930 nm (EB-NS), ≈ 950 nm (HB-NS) and ≈ 920 nm (HP-NS).

All three bulk starting materials showed a broad absorption in the green-red region of the visible spectrum (Fig. 3 and S17†). Similarly, the NIR fluorescence of EB, HB and HP showed the known NIR emission features, with HB displaying a red-shifted fluorescence (up to 1000 nm).^21–23^ This shift to longer wavelengths (Fig. 3b, e and S18†) opens possibilities in the field of *e.g.* biological imaging. Both excitation and emission features remained unaltered (Fig. 3d–f) in the NS, thus demonstrating that the exfoliation process of EB, HB and HP does not significantly alter the photophysics of these NIR fluorophores.

**Fig. 4:**
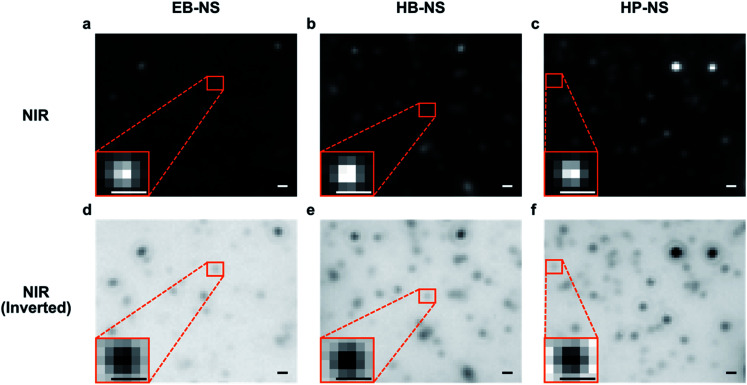
Near-infrared (NIR) imaging of single silicate nanosheets. Fluorescence images of EB-NS, HB-NS and HP-NS (*λ*_exc_ = 561 nm) in the NIR (> 900 nm). Most nanosheets appear as resolution-limited spots (Abbe limit ≈ *λ*_emi_/2). Note that some NS are so bright that they saturate the detector and appear to be larger than the resolution limit, but the AFM data proves otherwise (Fig. 2). Both normal (a–c) and inverted (d–f) grey scales are shown for better clarity. Scale bar = 1 μm.

The fluorescence of these NS can also be detected/imaged on the single particle level in a custom-built NIR fluorescence microscope. Imaging experiments showed that the NS are relatively monodisperse, with a size below the resolution limit (*d* < 500 nm) of light microscopy (Fig. 4). Furthermore, all NS displayed no photobleaching during 2 h-long laser illumination (≈ 200 mW effective laser power) and imaging (Fig. S19†). However, some of the NS were so bright that they saturated the local pixels and looked ‘larger’ than the resolution limit. This finding could be explained by either differences in size (see AFM data) or agglomeration of multiple NS within resolution-limited (500 × 500 nm^2^) regions. The question of how fluorescence scales with size is still not well understood but it likely increases with volume and requires further investigations.^17^

### Fluorescence lifetime measurements in the time and frequency domains

**Fig. 5:**
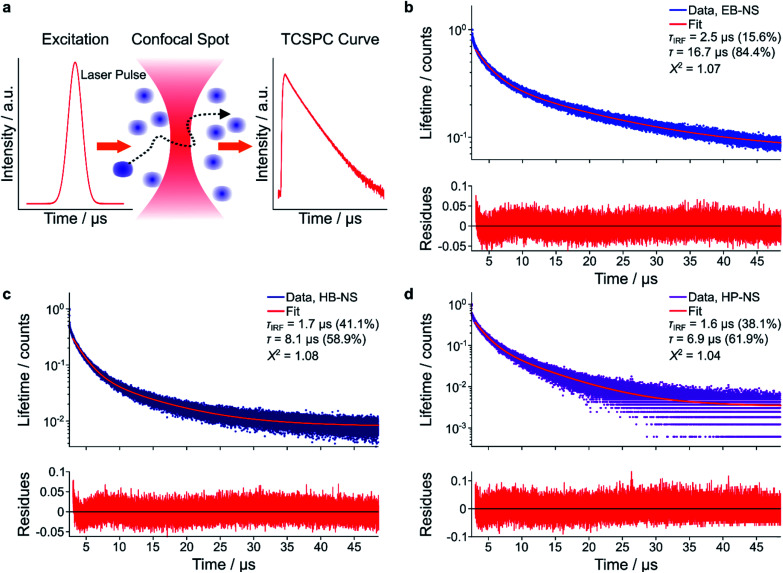
Fluorescence lifetimes of silicate nanosheets. (a) Schematic of the confocal time-correlated single-photon counting (TCSPC)-based measurements of fluorescent lifetimes of single NS diffusing freely in a solution. (b–d) Exemplary decay curves of EB-NS (b), HB-NS (c) and HP-NS (d), and the corresponding bi-exponential fits. Note that the short lifetime component corresponds to the instrument response function. Fit residues are given below the plot.

As discussed above, bulk EB presents a high quantum yield and a long fluorescence lifetime (*τ*), which is the consequence of a parity-forbidden electronic transition of the copper ion.^22,23,25^ To better understand what affects fluorescence lifetimes, they were measured with complementary methods. First, we used time-correlated single-photon counting (TCSPC) in a confocal setup (MicroTime 200, Picoquant) (Fig. 5a–d). We noticed that a single-exponential decay did not well describe TCSPC curves for all NS types (Fig. S20†). We therefore attributed the shorter component to the laser pulses (Instrument Response Function, IRF). The (longer) fluorescence lifetimes were determined to be 16.5 ± 0.25 μs, 8.25 ± 0.15 μs and 9.91 ± 0.06 μs for EB-NS, HB-NS and HP-NS, respectively.

This decrease in fluorescence lifetime from bulk (EB ≈ 130 μs, HB ≈ 60 μs, HP ≈ 25 μs) to NS was in agreement with frequency-domain measurements (Fig. S21†). An explanation for the observed decrease could be that defects or changes in symmetry either enable non-radiative decay pathways or increase the radiative rate constants.

### Microscopic and macroscopic fluorescence lifetime imaging (FLIM) of nanosheets

Fluorescence lifetimes are very sensitive measurements for the environment and can also be used for multiplexing. The μs lifetimes of silicate NS are an interesting property because they are orders of magnitude longer than typical organic fluorophores (which are in the nanoseconds range).

**Fig. 6:**
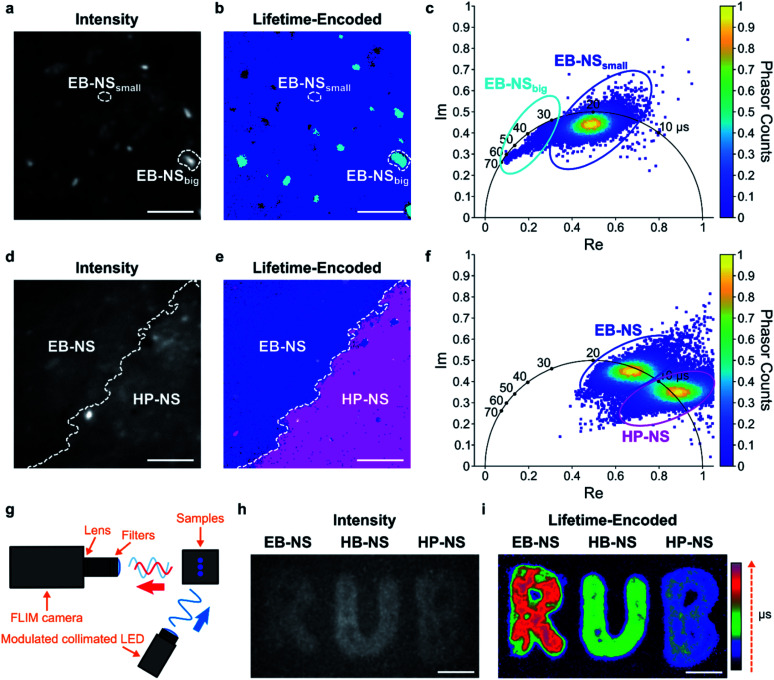
Fluorescence lifetime imaging of nanosheets. (a) Fluorescence intensity image of an EB-NS solution drop-casted on a glass coverslip. Scale bar = 5 μm. (b) Frequency-domain fluorescence lifetime imaging microscopy (FLIM) of the same region as in (a), but with a color-coding that shows two sub-populations of different lifetime. Scale bar = 5 μm. (c) Corresponding phasor plot highlighting the two populations and the color-coding in (b). Species with longer lifetime appear in a light blue color and the ones with the shorter lifetime appear in a dark blue color. (d) Fluorescence intensity image of the border of stripes made from EB-NS and HP-NS. Scale bar = 5 μm. (e) FLIM image of the same region as in (d), but with a color-coding that shows the different NS according to their lifetime values. Scale bar = 5 μm. (f) Phasor plot highlighting the color-coding of the lifetimes in (e): EB-NS (longer lifetimes, blue color) and HP-NS (shorter lifetimes, purple). (g) Schematic of the macroscopic frequency-domain FLIM setup. (h) Intensity image of concentrated NS drop-casted on paper showing the logo of the Ruhr-Universität Bochum (RUB). Scale bar = 5 mm. (i) FLIM image of the same piece of paper as in (h), but with lifetime-encoded colors of EB-NS, HB-NS and HP-NS. Scale bar = 5 mm.

Therefore, we built a setup for frequency-domain fluorescence lifetime imaging microscopy (FLIM).^56,57^ This combination of FLIM and wide-field microscopy allows for fast FLIM with variable optical magnification. In short, the laser excitation is modulated, and from the delay of the fluorescence signal one can infer the lifetime. This way one can directly receive a fluorescence lifetime image in contrast to scanning methods that have to measure lifetimes pixel by pixel. However, the necessary time-gated camera for frequency domain measurements required a careful calibration using samples with known lifetime values. Therefore, an independent measurement of single NS lifetime was required. For calibrating the frequency domain-based lifetime camera, we used lifetime values of EB-NS obtained with the TCSPC-based method described above (Fig. 5b), and imaged a drop-casted EB-NS in a homogeneous layer on top of a glass coverslip. Then, we imaged non-homogeneous layers of all NS types (Fig. 6 and S22†).

Furthermore, due to different lifetimes of EB-NS and HP-NS, we could identify the NS type (border) based on a lifetime image of a region with both EB-NS and HP-NS (Fig. 6d–f). This identification was performed by using the corresponding phasor plot (see Materials and methods†). A phasor plot represents frequency-domain lifetime data using the measured phase angle (*φ*) and the modulation ratio (*M*), calculated for each single camera pixel and defined as:

1 Im = *M* × sin(*φ*),

2 Re = *M* × cos(*φ*).

In our case, on such a phasor plot we could highlight two sub-populations of NS (blue and purple ovals in Fig. 6f). Then, we applied a color selection to the FLIM image (Fig. 6e). When only using the intensity image, it was not possible to identify the border between EB-NS and HP-NS (Fig. 6d). The average lifetimes of all NS types deduced from the lifetime images are: 17.6 ± 2.4 μs, 7.8 ± 1.5 μs and 4.5 ± 0.7 μs for EB-NS, HB-NS and HP-NS, respectively. Moreover, we noticed that, for each NS type, aggregates and particles with larger size exhibit slightly different lifetimes (Fig. 6a–c, S22 and S23†). In addition to microscopic FLIM, the high brightness of NS enabled macroscopic lifetime imaging (Fig. 6g). Indeed, drop-casting of ethanol dispersions of EB-NS, HB-NS and HP-NS on paper enabled us to detect their fluorescence (Fig. 6h), and to clearly distinguish them according to their uncalibrated phase lifetime (Fig. 6i). In this way, by using the NS as an ink we could encode the letters of the logo of the Ruhr-University Bochum (RUB) as lifetimes. These experiments show the huge potential for NIR FLIM and how it drastically improves the contrast, which is desirable for many imaging applications.

The lifetime values obtained using TCSPC were in good agreement with the lifetime values measured by frequency-domain FLIM (Tables 1 and S1†). Furthermore, the lifetime values measured with a simple fiber-optic sensor were in the same range, too (Table S1†). Therefore, the μs fluorescence lifetimes of these NS can be detected by methods of different complexity and potential.

**Table 1:** Fluorescence lifetime values of EB-NS, HB-NS and HP-NS measured using confocal TCSPC-based technique^a^

Sample	*τ*_TCSPC_ [μs]
EB-NS	16.50 ± 0.25
HB-NS	8.25 ± 0.15
HP-NS	6.91 ± 0.06

a Mean ± standard deviation (*N* = 3–4 measurements).

### Remote imaging through tissue

**Fig. 7:**
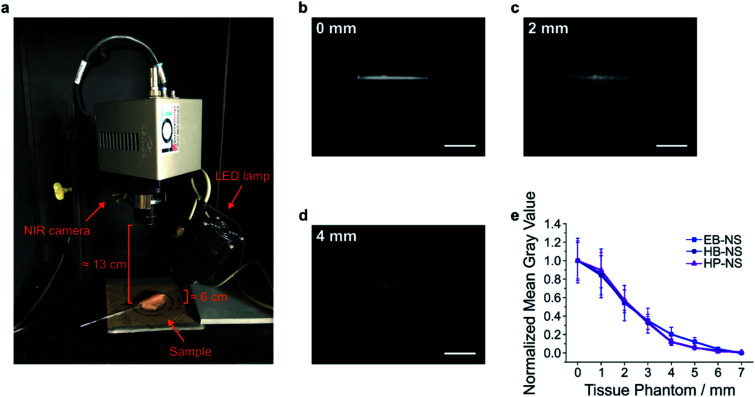
Remote NIR imaging of nanosheets under tissue. (a) Picture of the NIR remote (stand-off) detection system. Capillary tubes (containing NS in water) under chicken tissue phantoms were imaged as shown in the image. (b–d) NIR image of capillary tubes filled with EB-NS under a chicken tissue of a certain thickness. Scale bar = 10 mm. (e) Normalized mean gray value of a region of interest within each image *vs.* tissue phantom thickness. Error bar = standard deviation, normalized over the maximum value of mean pixel intensity within a ROI (area ≈ 19 × 1 mm^2^) from one image.

In a next step, we explored the potential of the NS in remote (stand-off) imaging through a tissue phantom. In such experiments, NS are imaged in a distance > 10 cm without a sophisticated optical setup or microscope (Fig. S24†). It mimics the requirements *e.g.* for image-guided surgery or remote imaging of fluorescent barcodes. We observed that the NS displayed a strong fluorescence sufficient for stand-off detection (Fig. S25–S28†). Furthermore, by monitoring the fluorescence intensity of NS solutions after addition of salts or lowering the pH, we noticed no significant decrease in the fluorescent signal (Fig. S8–S11†). Proven that these NS possess a robust and stable fluorescence, we performed a proof-of-principle experiment for biological imaging *e.g.* of vessels, by introducing EB-NS, HB-NS and HP-NS into glass capillary tubes (Fig. 7a and b).

Their intense fluorescence can be detected without a laser, *i.e.* with a simple LED (equipped with a 700 nm short-pass filter) and with a 900 nm long-pass filter in front of the NIR InGaAs camera. Next, we performed tissue phantom experiments, a standard test routinely performed in biomedical imaging (Fig. 7a and S29–S31†). The goal of this experiment was to assess the brightness of the sample through tissue slices of different thickness. Multiple 1 mm-thick chicken phantom tissues were therefore placed on top of NS-containing capillary tubes, and NIR pictures were taken (Fig. 7b and c). For each image, the intensity within a selected region of interest (ROI) was evaluated after every addition of a 1 mm-thick tissue slice. The fluorescence intensity of all silicate NS showed a similar trend (EB-NS seemed to display a slightly better performance) and was intense enough to be detected through up to 4–5 mm of tissue (Fig. 7d and e). Higher light intensities *e.g.* a laser illumination could further increase the tissue penetration. These results, together with the robustness of the fluorescence against biological analytes and pH ranges, and the high biocompatibility of EB-NS, HB-NS and HP-NS (Fig. S32–S34†), clearly demonstrate the potential of these NIR nanomaterials for biomedical imaging applications.

## Conclusions

In this work, we developed a robust protocol to exfoliate the layered silicates EB, HB and HP *via* a combination of planetary ball milling, tip sonication, and multi-step centrifugation, obtaining nanosheets of high monodispersity (lateral size ≈ 16–27 nm and height ≈ 1–4 nm). Thorough characterization *via* AFM and SEM allowed a complete description of the morphology of these NS. In the future, the exfoliation could be further improved by using surfactants that facilitate faster exfoliation and reduction of the required mechanical forces (milling/tip sonication).

Most importantly, EB-NS, HB-NS and HP-NS retained the absorption (≈ 550–650 nm) and emission (≈ 850–1000 nm) properties of the non-exfoliated material. Therefore these NS are an addition to the library of NIR fluorescent nanomaterials beyond ICG, InAs quantum dots, lanthanide-doped nanoparticles and SWCNTs.^44–49^ The fluorescence of all three NS is very bright, and single nanosheets can be easily imaged with a NIR fluorescence microscope. Therefore, single nanoparticle fluorescence applications such as microrheology or, in the future, chemical sensing^52,58–60^ should be possible.

Interestingly, the fluorescence lifetimes decrease in comparison to bulk silicates, especially for EB-NS. This result indicates that lifetimes of these nanomaterials can be tailored and be used for lifetime-encoded imaging.^47^ Microscopic fluorescence lifetime imaging can result in increased contrast, considering that short-lived background fluorescence can be easily removed. Furthermore, FLIM presents several advantages over standard fluorescence intensity imaging including independence from fluorescence intensity, fluorophore concentration or bleaching. Additionally, the fluorescence lifetime can be sensitive to environmental factors, which could facilitate the monitoring of functional changes in biological systems.^61^ Macroscopic fluorescence lifetime imaging experiments demonstrated an additional benefit of working with such bright and robust materials, *i.e.* that they can be clearly detected and unambiguously distinguished even when using non-optimized home-built stand-off devices. The μs fluorescence lifetimes do indeed not require complicated setups as normally needed for ps- to ns-long lifetimes of other typical NIR fluorophores (*e.g.* SWCNTs). For this reason, the successful application of such NS for barcoding *e.g.* banknotes but also biological materials or even cells is within reach. The advantage of the NS' intense fluorescence was also shown by stand-off detection experiments with tissue phantoms. Considering their high biocompatibility, these NS have large potential for bioimaging.

In summary, exfoliated EB-NS, HB-NS and HP-NS possess versatile NIR fluorescence properties and pave the way for novel fluorescence (lifetime) bioimaging applications and for photonics in general.

## Experimental

### Exfoliation of Egyptian blue (EB), Han blue (HB) and Han purple (HP) bulk powders into nanosheets (EB-NS, HB-NS and HP-NS, respectively)

EB, HB and HP powders (respectively < 120 μm, < 40 μm and < 40 μm qualities) were purchased from Kremer Pigmente GmbH & Co. KG. For each powder, exfoliation was performed as follows.

In the milling step, 3 g of powder were introduced into a 20 mL agate beaker together with 5 mm agate balls. Deionized water was added until a slurry consistency was reached. The so-prepared agate beaker was then placed in a planetary ball mill (PB, Pulverisette 7 Premium Line, Fritsch), which was run at 900 rpm for 1 h (3 cycles of 20 min each, 5 min pause). Aliquots of the resulting milled slurry were removed from the beaker and their grain size distributions were measured by means of a laser diffraction particle sizer (LDPS, model LS13320, Beckman&Coulter). For each measurement, three runs were performed, PIDS was used, and an optical model R.I. 1.6/1 was applied. To visualize the grain size distribution, the number of particles within an individual grain size class was plotted as percentage of all particles against the central diameter of the class. In this way, the very small number of bigger particles that was present in the system does not dominate the distribution curve, as it would instead happen if volume percentages were displayed.

Following the milling step, an aliquot was poured into a Nalgene® centrifuge tube (Thermo Fisher Scientific) and water was added until an overall volume of 150 mL (dilution factor ≈ 3) was reached. Then, a first centrifugation step (Heraeus Multifuge X3R, Thermo Fisher Scientific) was performed: *T* = 20 °C, 800 rpm (150 × *g*), 9 min 41 s, 5 s acceleration ramp, 5 s deceleration ramp, 5 cycles. These parameters were calculated from the Stokes equation (corrected for centrifugation^62^) in order to remove particles of diameter *d* > 1 μm:3
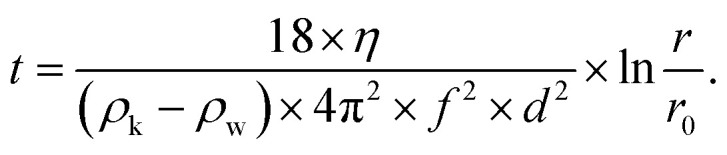
In eqn (3), *t* is the settling time [s], *η* the temperature-dependent dynamic viscosity of water [kg m^−1^ s^−1^], *ρ*_k_ the grain density [kg m^−3^], *ρ*_w_ the temperature-dependent water density [kg m^−3^], *f* the rpm [s^−1^], *d* the previously mentioned grain (equivalent) diameter [m], *r* the distance between the rotor's fulcrum and the sediment's height [cm], and *r*_0_ the distance between the rotor's fulcrum and the suspension's surface [cm] (the last two parameters are dependent on the geometry of the employed centrifuge). At the end of each cycle, the supernatant was decanted and new water was added up to the initial volume; finally, the so-obtained supernatant dispersion (pH ≈ 10–11) was collected in a glass jar and stored at room temperature. New LDPS measurements were performed to check the efficiency of the described centrifugation step in terms of the size distribution. Aliquots of each supernatant of each of the three milled pigments were taken, dried and weighed, yielding the following concentrations: EB ≈ 2.2 g L^−1^, HB ≈ 1.4 g L^−1^ and HP ≈ 0.6 g L^−1^. While EB proved to be stable for up to ≈ 2 days already at this stage, milled HB and HP particles settled after a few hours.

The supernatant vials were vigorously shaken and specific volumes of them were withdrawn for the following steps. More precisely, in order to carry out the second exfoliation procedure of each silicate in a total volume of 50 mL with comparable concentrations, sample aliquots were diluted with additional Milli-Q water until the lowest concentration among the three samples (*i.e.* HP ≈ 0.6 g L^−1^) could be reached in all systems. Typically, a so-diluted milled supernatant was then poured into a 50 mL glass vial and subjected to tip sonication (Fisherbrand™ Model 120 Sonic Dismembrator, Fisher Scientific) in an ice bath for 6 h at 60% amplitude (*i.e.* 72 W).

A second and last centrifugation step followed, necessary to remove metal residues from the previous tip sonication step. Typically, the sample was introduced into a 50 mL Falcon tube, which was then centrifuged under the following conditions: *T* = 20 °C, 1500 rpm (526 × *g*), 4 min 12 s, 9 s acceleration ramp, 7 s deceleration ramp, 1 cycle. While most of the sample's volume could be efficiently decanted in this way, an additional step at 3000 rpm (2103 × *g*) for 1 min was performed on its last fraction in the Falcon tube (<5 mL), where the removal of the supernatant from the sediment was typically more challenging. Here also, parameters were optimized to remove particles larger than 1 μm.

The so-generated samples (EB-NS, HB-NS and HP-NS) had a pH ≈ 8–9, concentration of ≈ 0.5 g L^−1^, measured by drying aliquots of known volume. The final sample vials were in conclusion stored at room temperature. Further characterization steps were always preceded by either vortexing (Vortex Mixer VV3, VWR International) at maximum power or by bath sonication (Branson 1800 Cleaner, Sonics Online) for 10 min. Unless explicitly stated otherwise, measurements described in this paper were performed in the same way for EB-NS, HB-NS and HP-NS, which are therefore referred to as “NS”.

### Atomic force microscopy (AFM)

A typical NS sample was vortexed and bath sonicated for 10 min, then 5–10 μL of undiluted solution were spin-coated (G3 Spin Coater, Specialty Coating Systems, Inc.) onto a previously Scotch-tape exfoliated mica surface according to the following parameters: 10 s of ramp time, 2000 rpm, 150 s of dwell time. An Asylum Research MFP-3D Infinity AFM (Oxford Instruments) was employed in AC mode (software version 15.01.103). Rectangular cantilevers from Opus (160AC-NA, MikroMasch Europe) were used: these were characterized by aluminum coating, a tetrahedral tip, 300 kHz resonance frequency, and a force constant of 26 N m^−1^. For the statistical analysis, sample regions with sizes of 5 × 5 μm^2^ were measured at 1.03 Hz and with 512 points per line (which corresponds to 9.8 nm of pixel size). High resolution AFM images were, on the other side, acquired on sample regions of 1 × 1 μm^2^, at 0.85 Hz and with 1024 points per line (977.5 pm of pixel size). Image analysis was carried out with the software Gwyddion (version 2.51). For size distribution estimations, the mean diameter (*i.e.* mean radius *R*_m_ × 2 = *D*_m_) and the maximum height (*z*_max_) obtainable in Gwyddion after height and slope thresholding were considered. Python scripts based on the module scipy.stats were run for the fitting of histograms and scatter plots: the former class of fits are log-normal distribution functions, typical for fragmentation phenomena like the ones endured by our NS with our protocols,^55^ whereas the latter is represented by linear fits. The parameters of the histograms were calculated using a maximum likelihood estimation method.

### Scanning electron microscopy (SEM) and scanning transmission electron microscopy (STEM)

For SEM imaging (Quattro S SEM, Thermo Fisher Scientific), highly-oriented pyrolytic graphite (HOPG, grade ZYB, Bruker) were used as substrates. HOPGs were plasma-treated (Zepto Diener Electronic GmbH +Co. KG, 1 min of O_2_ supply, 1 min of plasma process) in order to clean their surfaces and increase their hydrophilicity. Next, a typical NS sample was vortexed and bath sonicated for 10 min. 10 μL of undiluted sample were spin-coated with the same parameters employed for AFM measurements. For each NS sample, the so-prepared HOPG was either imaged at the SEM as is, or a ≈ 4 nm-thick gold layer was evaporated (Baltec MED-020, Baltec) onto it to decrease surface charging and, thus, increase imaging contrast. The HOPG was then placed into the SEM chamber and imaged in the following conditions: high vacuum mode, voltage = 5.00 kV, spot size = 3.0, working distance = 10.0 mm, Everhart–Thornley Detector (ETD) for secondary electrons, Circular Backscatter Detector (CBS) for backscattered electrons.

STEM measurements were carried out on the same device. Typically, undiluted 5 μL of previously vortexed and bath sonicated NS were deposited and dried onto formvar-coated copper grids, stabilized with evaporated carbon film (FCF300-CU, Electron Microscopy Sciences). The parameters are the same as for SEM imaging, except for the employed detector (STEM3+).

### Near-infrared (NIR) imaging at microscopy setup

The imaging setup consists of a 561 nm laser (Cobolt Jive™ 561 nm), an Olympus IX53 microscope equipped with a 100× (UPlanSApo 100×/1.35 Sil, Olympus) and a 20× (MPlanFL N 20×/0.45, Olympus) objectives, and a NIR camera (Cheetah TE1, Xenics). Along the light path leading from the microscope to the camera, a dichroic mirror (VIS/NIR, HC BS R785 lambda/5 PV, F38–785S, AHF) and a 900 nm long-pass filter (FELH0900, Thorlabs) are installed. NS droplets were imaged directly after drying on a #1 glass coverslip.

For simple characterization images, a 5 μL droplet (≈ 0.5 g L^−1^) of heavily vortexed NS was drop-casted on the imaging substrate; acquisition was performed with the 100× objective, an integration time of 0.5 s and the laser was set at an excitation power of 250 mW.

To investigate whether NS can be bleached, a long imaging session with continuous excitation was performed on 10 μL of NS sample. Acquisition settings were the following: objective = 20×, laser set power = 500 mW, measured power out of the objective ≈ 180 mW, acquisition time ≈ 2 h, exposure time = 0.5 s, frame rate = 0.3 fps.

Data acquisition was controlled by the Xenics software (v. 2.6), whereas data analysis was carried out in ImageJ (v. 1.52a) and on Origin Pro 8.1. Background subtraction (*i.e.* subtraction of dark regions within the original images) was also performed in ImageJ, but was only applied to Fig. 4.

### Near-infrared (NIR) 1D and 2D spectroscopy

The spectroscopy setup presents two different light sources: a laser (Gem 561, Laser Quantum, Novanta) and a monochromator (MSH-150, LOT-Quantum Design GmbH, equipped with a xenon arc lamp and a diffraction grating). While the former was employed for the study of 1D emission profiles, the latter was used for acquisition of 2D spectra. The microscope is an Olympus IX73 with a 10× objective (UplanFLN 10×/0.30, Olympus), whereas the spectrometer is a Shamrock 193i spectrograph (Andor Technology Ltd.) coupled to an array NIR detector (Andor iDUs InGaAs 491). NS (300 μL, undiluted) were introduced into a 96-well-plate, positioned above the setup's objective; ≈ 1–3 mg of bulk (pristine) silicate powders were dispersed in the same Milli-Q water volume and measured, too. Fluorescence data was acquired *via* the Andor SOLIS software (version 4.29.30012.0).

For the 1D dataset, the exposure time was set to 1 s, the laser power to 100 mW, and the input side slit width to 500 μm. Data analysis and plotting were performed by means of the software Origin Pro 8.1; more precisely, the peak positions were evaluated *via* 1^st^ derivative method with Savitzky–Golay smoothing (Peak Analyzer-Integrate Peaks, polynomial order = 2, points of window = 20). Full widths at half maximum (FWHM) were automatically assessed by the software at the end of this procedure.

For acquisition of 2D spectra, the monochromator light source was scanned with steps of 5 nm over a wavelength range of 400–700 nm; during a single measurement, a spectrum was recorded with an integration time of 4 s and 10 s for bulk and NS samples, respectively (input side slit width = 500 μm). The plotting as well as the correction for the quantum efficiency of the detector and for the spectral irradiance of the xenon lamp were performed using a self-written Python script.

### Fluorescence lifetime measurements with time-correlated single-photon counting device

Confocal fluorescence lifetime measurements (Fig. 5a) were performed using the commercial confocal setup Microtime 200 (PicoQuant GmbH). The system is based on an Olympus IX-71 inverted microscope (Olympus Deutschland) with a side-port on the right side. The excitation unit consists of a pulsed diode laser (*λ*_exc_ = 640 nm, LDH-D-C-640, PicoQuant GmbH) with a repetition rate of 80 MHz. To ensure enough excitation efficiency, 100 pulses were sent together consecutively within a time of 1.25 μs with a subsequent laser-off time of 48.75 μs, during which emitted photons were collected. The total acquisition time for each measurement was 10 min. The laser power was maintained at 20 μW in the back-focal plane of the objective lens. A high-NA objective (UApoN 100× oil, 1.49 NA, Olympus) was used to focus the light inside the sample and at the same time to collect the emission photons. Collected emission light was passed through the dichroic mirror (Di01-R405/488/561/635, Semrock) and focused through a pinhole (diameter = 100 μm) for confocal detection. After the pinhole, the light was refocused onto an avalanche single photon diode (τ-SPAD, PicoQuant) using two achromatic lens doublets. A long-pass filter (647LP, Semrock) was used to block backscattered excitation light. Signals from the detector were processed by a multi-channel picosecond event timer (Hydraharp 400, PicoQuant) with 16 ps time resolution.

Analysis of fluorescence lifetime decays was performed using a custom-written MATLAB routine. First, photon arrival times were read from the raw.hdd data files with MATLAB functions provided by PicoQuant. Single- and double-exponential decay functions were then used to tail-fit the TCSPC curves, within a time window from 2.6 μs till 50 μs after excitation pulse (Fig. S20† and 5b–d).

### Fluorescence lifetime measurements with fiber-optic oxygen sensor

The chosen fiber-optic oxygen sensor (FireSting O_2_, PyroScience GmbH) is equipped with a fluorophore (REDFLASH indicator) which is excitable at orange-red wavelengths ≈ 610–630 nm and displays an oxygen-dependent luminescence at ≈ 760–790 nm. It was used for measurements in the frequency domain (Fig. S21a†) to determine fluorescence lifetimes. Measurements were performed on dry samples: while the bulk powders were measured without prior treatment, 10 mL of NS counterparts were freeze-dried over 2 days, yielding ≈ 10–15 mg of powder. Typically, the FireSting optical probe was lowered into a Falcon tube where few mg of sample powder were positioned; the fiber was then mechanically fixed at a distance from the sample which could result in the desired signal value (intensity ≈ 14 mV, constant for all samples) with our settings (*ν* = 4000 Hz, LED intensity = 60%, amplification factor = 400×). Each measurement took 10 min, while data points were acquired every second; signal (and calculated lifetime) values were showing a neglectable fluctuation, thus leading to a low standard deviation (not observable by eye in Fig. S21b†). However, given the overall low signal strength when measuring with this device, the TCSPC values should be regarded as the gold-standard.

### Microscopic frequency-domain fluorescence lifetime imaging

Measurements were performed using a custom-built optical setup (Fig. S35†). Light of an excitation laser with 638 nm wavelength (PhoxX+ 638-150, Omicron) was modulated with square-shaped pulses at 8 kHz frequency. Gating signal was sent by a camera to laser in order to switch it off during the camera readout time. A clean-up filter (CUF) (ZET 640/10, Chroma) was used to reject undesired wavelengths. A variable neutral density filter (ND) (NDC-50C-4-A, Thorlabs) was used for adjusting the laser power. Afterwards, the laser light was coupled into a single-mode optical fiber (SMF) (P1-460B-FC-2, Thorlabs) with a typical coupling efficiency of 50%. After exiting the fiber, the light beam was expanded by a factor of 3.6 using two telescopic lenses (TL1 and TL2). The collimated laser light was focused into the back focal plane of the objective (UAPON 100× oil, 1.49 NA, Olympus) using lens L1 (AC508-180-AB, Thorlabs). For reducing back-reflections of the excitation light, the illumination angle on the sample was varied slightly by mechanically shifting the collimated beam with a translation stage TS (LNR50M, Thorlabs) perpendicular to the optical axis. A high-performance two-axis linear stage (M-406, Newport) ensured smooth lateral sample positioning. An independent one-dimensional translation stage (LNR25/M, Thorlabs) was equipped with a differential micrometer screw (DRV3, Thorlabs) to move the objective along the optical axis for focusing. Collected fluorescence was spectrally separated from the excitation path using a multi-band dichroic mirror (DM) (Di03 R405/488/532/635, Semrock) that guided the fluorescence light towards the tube lens L2 (AC254-200-A-ML, Thorlabs). Lenses L5 (AC254-100-A, Thorlabs) and L6 (AC508-150-A-ML, Thorlabs) re-imaged the fluorescence from the image plane located after the tube lens onto the chip of an emCCD camera (iXon Ultra 897, Andor). Alternatively, lenses L3 (AC254-100-A, Thorlabs) and L4 (AC508-075-A-ML, Thorlabs) re-imaged the light onto the chip of a lifetime camera (PCO.FLIM, PCO AG). For switching the light between the two cameras, a dielectric mirror (BB1-E02, Thorlabs) was positioned on a magnetic base plate MB (KB50/M, Thorlabs) with a removable top. A band-pass filter BP (BrightLine HC 692/40, Semrock) was used to reject scattered excitation light in the emCCD detection path. Alternatively, a long-pass filter LPF (647 nm EdgeBasic, Semrock) was used for the frequency domain-based lifetime camera path. The magnification for imaging with the emCCD was 166.6×, resulting in an effective pixel size in sample space of 103.5 nm. The magnification for imaging with the lifetime camera was 83×, so that the effective pixel size in sample space was 84 nm. The total acquisition time for a single frame with lifetime information was 3.2 s and consisted of 16 consecutive sub-frames with exposure times of 200 ms each. Careful calibration was required in order to obtain absolute lifetime values using the frequency domain-based lifetime camera. The sample with an homogeneous layer of EB-NS drop-casted on top of a glass coverslip was used for calibration. The lifetime value used as a reference for calibration was obtained using a confocal TCSPC-based measurement of the same EB-NS sample. The excitation laser power was in the range of 20–30 mW.

### Macroscopic frequency-domain fluorescence lifetime imaging

The macroscopic lifetime imaging setup (Fig. 6g) consists of the above mentioned PCO.FLIM camera, a lime-colored LED, and a custom-built stage to hold the sample in place. The camera is positioned at a distance of ≈ 30 cm from the sample, and is equipped with a fixed focal length objective (*f* = 16 mm/F1.4, Thorlabs), an 800 nm long-pass filter (FEL0800, Thorlabs), and a 920 nm band-pass filter (FB920-10, Thorlabs). The LED illuminates the sample from a distance of ≈ 15 cm with a power below 1 W (M565L3, Thorlabs). The light beam is collimated by an aspheric condenser lens (ACL2520U-A, Thorlabs) and controlled by an LED Driver (LEDD1B, Thorlabs). During data acquisition, the LED was modulated by the camera with a sine wave of 5 kHz frequency and was not turned off during pixel readout. To achieve the best possible image quality, an increased exposure time of 5 s for each of the 16 sub-frames was used and a median filter with a kernel size of 5 was applied to reduce salt-and-pepper-noise. No calibration was performed prior to the acquisition.

### NIR imaging at stand-off detection setup

Our NIR custom-made stand-off detection setup^18^ consists of a NIR InGaAs camera (XEVA, Xenics), a Kowa objective (*f* = 25 mm/F1.4), and a white light source (UHPLCC-01, UHP-LED-white, Prizmatix). The latter is equipped with a 700 nm short pass filter (FESH0700, Thorlabs) for excitation. Furthermore, a 900 nm long pass filter (FEL0900, Thorlabs) is mounted on the camera.

For imaging of EB, HB and HP water dispersions in glass vials, an exposure time of 3 s at maximum lamp intensity was employed.

Concerning sample preparation for tissue phantom experiments, 1 mL of NS batch was concentrated (Concentrator 5301, Eppendorf) for ≈ 2 h, yielding a volume of ≈ 0.1 mL with concentration of ≈ 5 g L^−1^. After ≈ 5 min of bath sonication, a capillary tube (ringcaps® 25 μL, Hirschmann Laborgeräte GmbH & Co. KG) was dipped into the NS vial, and NS were thus sucked into the glass tube due to capillary forces. For each class of silicates, a so-prepared capillary tube was positioned onto the setup's stage at ≈ 6 cm and ≈ 13 cm away from the excitation lamp and the detection camera, respectively (Fig. S24†). Initial reference images were acquired with 0.5 s of exposure time at maximum excitation intensity. Afterwards, ≈ 1 mm-thick chicken slices were step-wise laid on top of the capillary tube, and new images with the same settings were taken after every layer's deposition.

Colloidal stability experiments were performed in cuvettes on sample volumes of ≈ 2 mL. EB-NS, HB-NS and HP-NS had a starting pH value in the range of ≈ 8–9. Prior to imaging, NS were bath sonicated for 5 min. Then, buffer solutions of pH 4 and 7 were added to some NS cuvettes in the volumes necessary to reach pH 5 and pH 7, respectively. To study the impact of ions on the colloidal stability of the NS, 200 μL of a solution of NaCl (9 g L^−1^ a typical blood concentration) were added into other cuvettes. The effect of phosphate-buffered saline (PBS) was also evaluated by adding 200 μL of 10× PBS into separate cuvettes, leading to a final 1× PBS concentration in the samples. Finally, control samples consisted in the addition of 200 μL of H_2_O. Imaging was started shortly after the introduction of the mentioned aliquots in the cuvettes. Acquisition settings were: exposure time = 2 s, frame rate = 0.067 fps, LED excitation power = 50%, acquisition time ≈ 60 min, LED-cuvette distance ≈ 18 cm, camera-cuvette distance ≈ 20 cm. To assess the fluorescence intensity over time, the mean signal intensity in a central region of interest of each cuvette was measured (and normalized to the starting frame).

Except for colloidal stability experiments, background subtraction (*i.e.* subtraction of a background reference image) was performed on the acquired dataset. Data analysis of the obtained NIR pictures was carried out in ImageJ (v. 1.52a) and Origin Pro 8.1.

## Preprint

This manuscript has been uploaded to a preprint server (https://doi.org/10.26434/chemrxiv.13350728.v1).

## Author contributions

SK and GS conceived and designed the study. SK coordinated the study. VK and GS worked on the different exfoliation methods and studied the size distributions *via* LDPS. GS and MW collected and analyzed the AFM dataset. GS and IM performed SEM characterization. GS measured absorption, as well as 1D and 2D excitation and fluorescence spectra. GS acquired the NIR microscopy images. RN and GS carried out macroscopic (stand-off) fluorescence imaging experiments. RT, NO and JE performed fluorescence lifetime spectroscopy as well as microscopic FLIM. MW built the macroscopic FLIM setup and acquired the respective lifetime dataset. TO performed cytotoxicity tests.

## Conflicts of interest

There are no conflicts to declare.

## Supplementary Material

NA-003-D1NA00238D-s001
