# Supplementary material for: Photophysical properties and fluorescence lifetime imaging of exfoliated near-infrared fluorescent silicate nanosheets
Source: Nanoscale Adv. 2021 Jun 24;3(15):4541–53. doi: 10.1039/d1na00238d (PMC9419235; doi:10.1039/d1na00238d)
Supplement: NA-003-D1NA00238D-s001 [file NA-003-D1NA00238D-s001.pdf]

Electronic Supplementary Material (ESI) for Nanoscale Advances.
This journal is © The Royal Society of Chemistry 2021

Supplementary Information

Photophysical Properties and Fluorescence Lifetime Imaging of Exfoliated Near-Infrared Fluorescent Silicate Nanosheets

*Gabriele Selvaggio, Milan Weitzel, Nazar Oleksiievets, Tabea A. Oswald, Robert Nißler, Ingo Mey, Volker Karius, Jörg Enderlein, Roman Tsukanov, Sebastian Kruss**

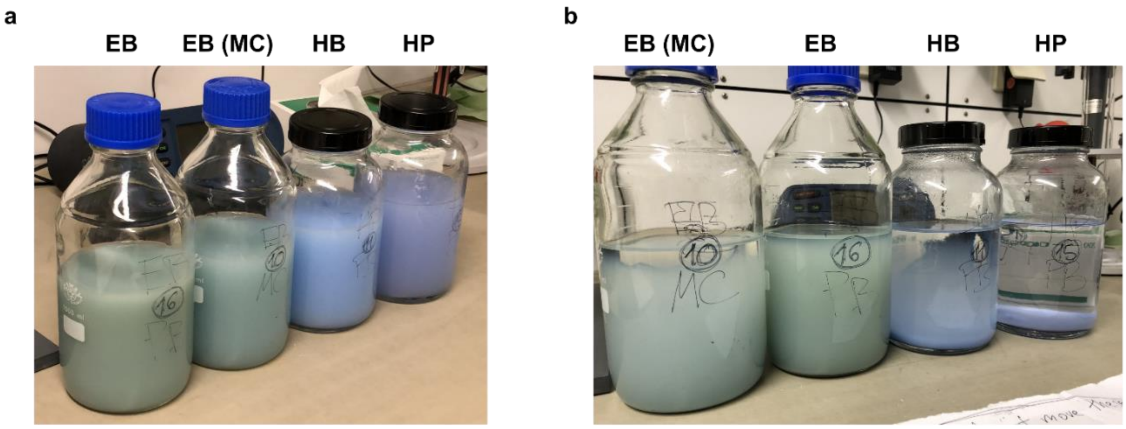

Figure S1. Colloidal stability of planetary ball (PB)/McCrone (MC) milled and centrifuged Egyptian Blue (EB), Han Blue (HB) and Han Purple (HP) a Picture of EB, HB and HP dispersions in water obtained after milling and centrifugation step #1. In addition to the standard PB technique, MC milling was also tested on EB. **b** Pictures of the same samples taken 3 days later to check the colloidal stability: while EB supernatants were still stable, HB and especially HP had started to settle down.

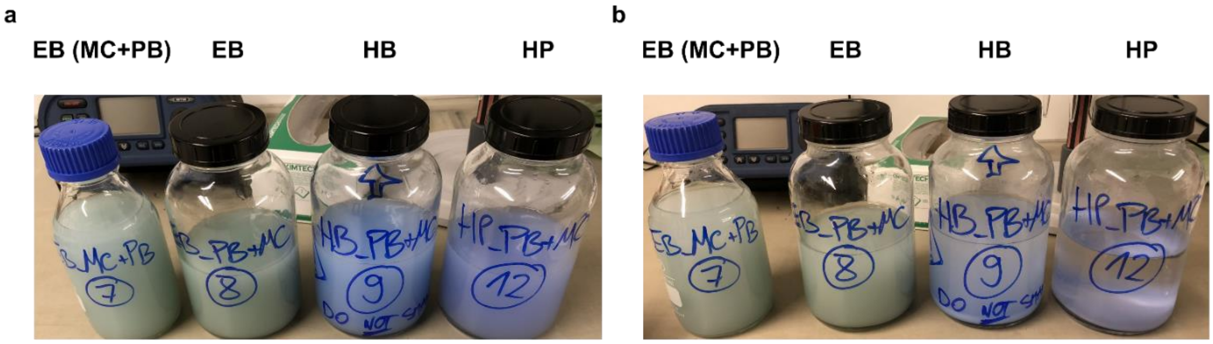

Figure S2. Colloidal stability of PB+MC/MC+PB milled and centrifuged EB, HB and HP Before choosing the planetary ball mill (PB) as best compromise for the milling step of EB, HB and HP, other techniques were tested. These included McCrone milling (MC) and mixed approaches (PB+MC, MC+PB). **a** Picture of EB, HB and HP dispersions in water obtained after mixed approaches of milling, followed by centrifugation step #1. For this dataset, MC+PB was performed only on EB. **b** Pictures of the same samples taken 1 day later to check the colloidal stability: while EB supernatants were still stable, HB and especially HP had started to settle down.

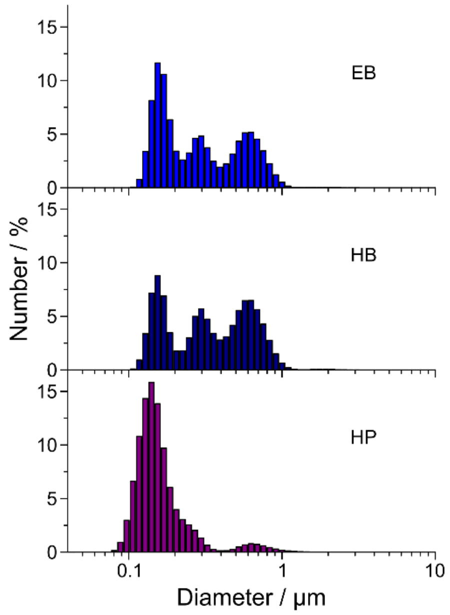

Figure S3. Size distribution of planetary ball milled (PB) EB, HB and HP after centrifugation step #1 (*i.e.* prior to tip sonication) Milled and centrifuged samples measured *via* laser diffraction particle sizer (LDPS). For EB, HB and HP alike, the efficiency of the centrifugation step is shown by the cut-off seen at ≈ 1 µm. All samples display a trimodal distribution, which is less pronounced in the case of HP. N = 1 independent sample per category, n = 3 measurement runs.

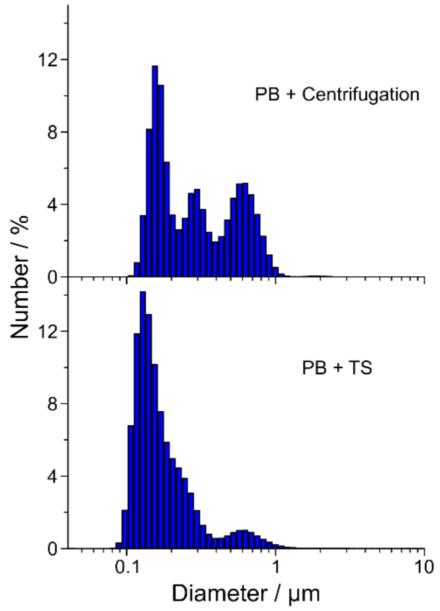

Figure S4. Effect of tip sonication (TS) on the size distribution of planetary ball milled (PB) EB Laser diffraction particle sizer (LDPS)-based analysis of only milled and milled + tip sonicated EB is presented. The former (milled) sample was centrifuged and its supernatant decanted before measurement, as described in the main manuscript text. The latter, on the other side, underwent a tip sonication step in addition to the milling one, and is then measured directly after exfoliation (as a whole, *i.e.* without centrifugation). When plotted in the form of histograms (number % *vs.* particle diameter), the LDPS dataset shows that the trimodal distribution is less pronounced after tip sonication, and its lowest extremes are shifted to lower diameter values, indicating a further size reduction. N = 1 independent sample per category, n = 3 measurement runs.

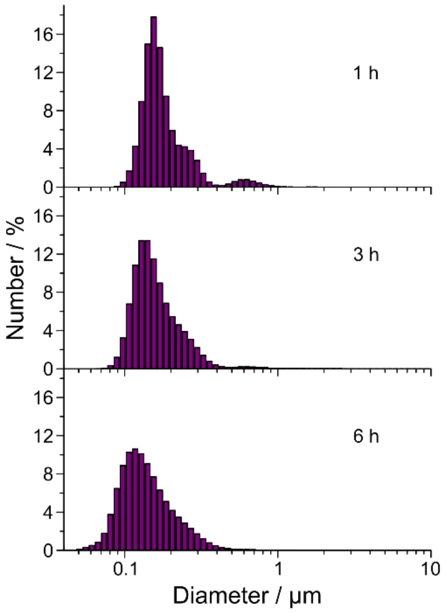

Figure S5. Monitoring of size distribution of Han Purple nanosheets (HP-NS) during tip sonication Laser diffraction particle sizer (LDPS)-based analysis shows the effect of tip sonication on the size distribution of a HP sample over time (1 h - 3 h - 6 h). When plotted in the form of histograms (number % *vs.* particle diameter), the LDPS dataset shows that the trimodal distribution gets less and less pronounced in time, and its lowest extremes are shifted to lower diameter values, indicating a further size reduction. $N = 1$ independent sample per category, $n = 3$ measurement runs.

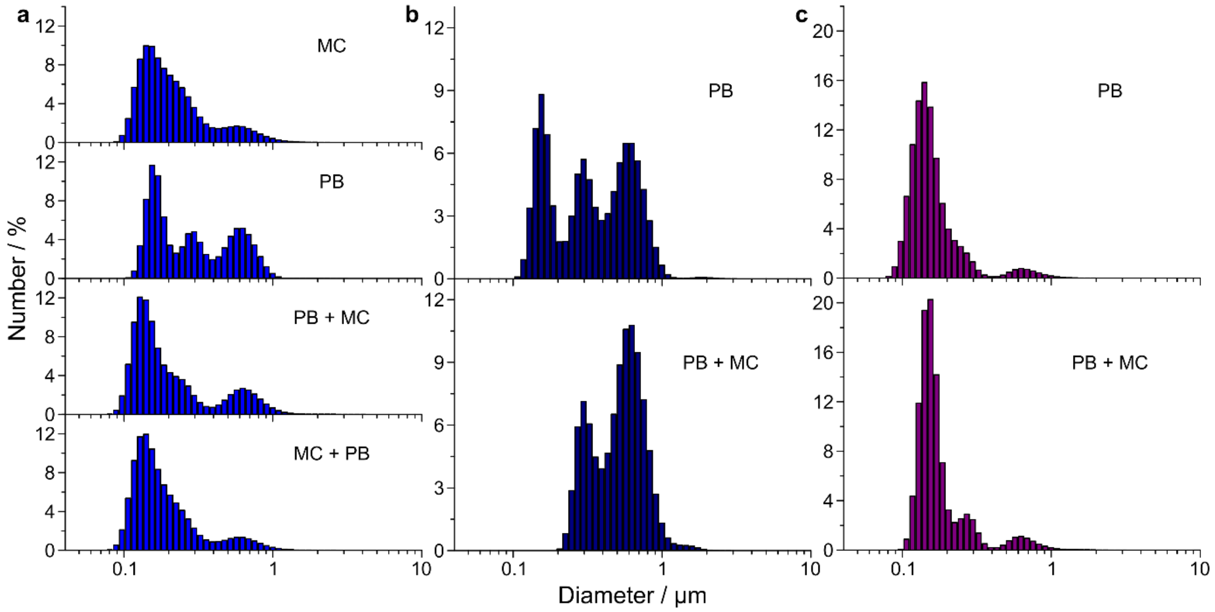

Figure S6. Comparison of exfoliation efficiency of different milling techniques Histograms (number % *vs.* particle diameter) obtained from a laser diffraction particle sizer (LDPS) show the obtained size distributions of EB (**a**), HB (**b**) and HP (**c**). Before measurement, all samples were centrifuged and the supernatant decanted. The efficiency of the employed centrifugation step is proven by the very low amount of particles larger than ≈ 1 µm. For EB, while the population's extremes seem to be consistent with all tested techniques, the trimodal distribution is more evident in the PB case. HB displays a much clearer difference between the tested PB and PB+MC; here, the former approach yields smaller particles and, thus, results more optimal. Finally, the HP sample does not show a pronounced difference between the previous two techniques. N = 1 independent sample per category, n = 3 measurement runs.

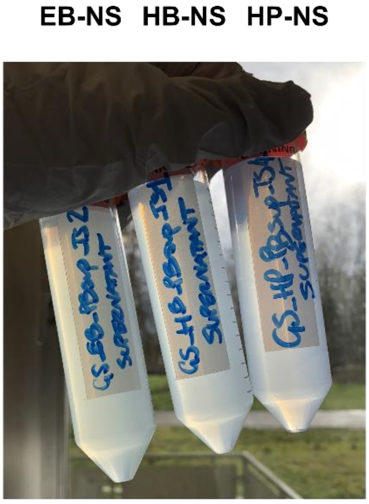

Figure S7. PB milled, tip sonicated and centrifuged EB-NS, HB-NS and HP-NS Self-taken photograph of the fully exfoliated samples, *i.e.* the final NS batches that underwent complete morphological and photophysical characterization.

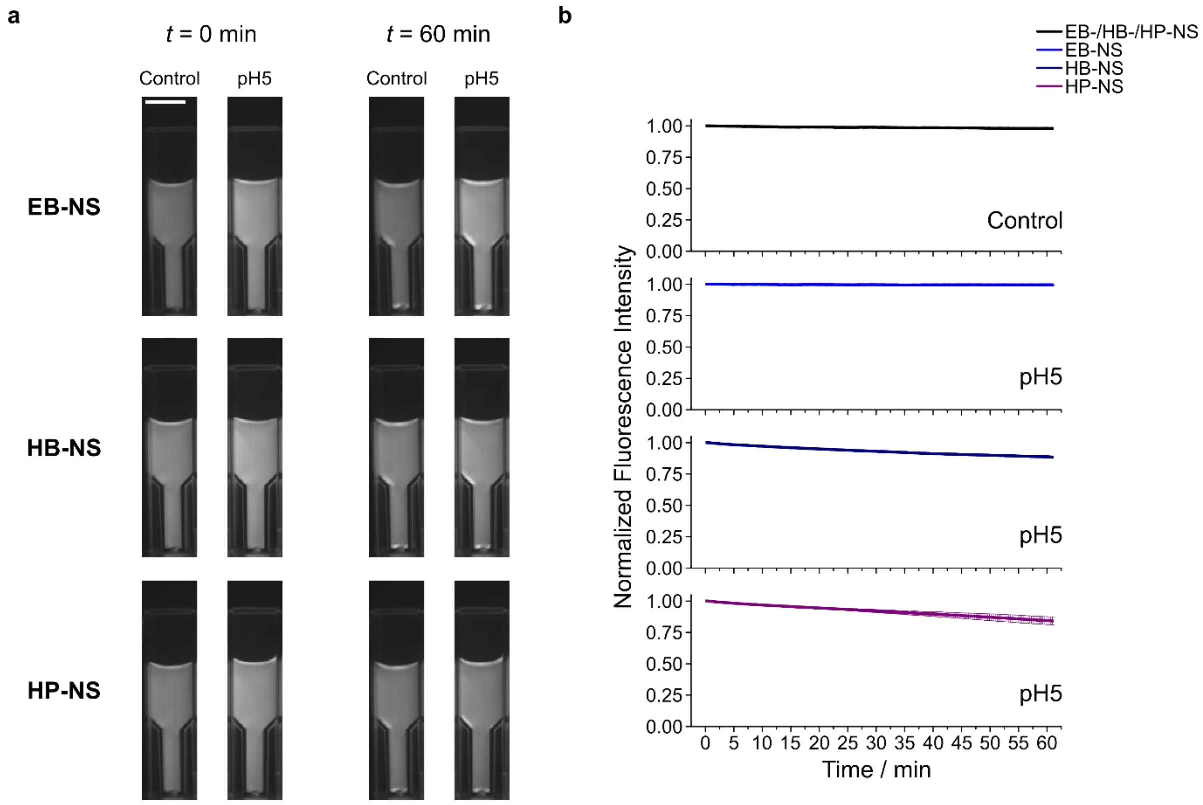

Figure S8. Colloidal stability of EB-NS, HB-NS and HP-NS in acidic conditions a Stand-off NIR fluorescence images of NS samples in cuvettes at pH 5. Measurements were taken over 1 h to assess the effect of acidic pH on the stability, *i.e.* the fluorescence of a central region of interest within the cuvette. Control = addition of H_2O (instead of buffer solutions). Scale bar = 1 cm. **b** Corresponding mean fluorescence intensity, normalized to the first frame. HB- and HP-NS performed worse than EB-NS. Nevertheless, all NS displayed a signal decrease not lower than 15%, thus proving to be significantly stable in such conditions. Error bars = standard deviation, $N = 2$ independent samples.

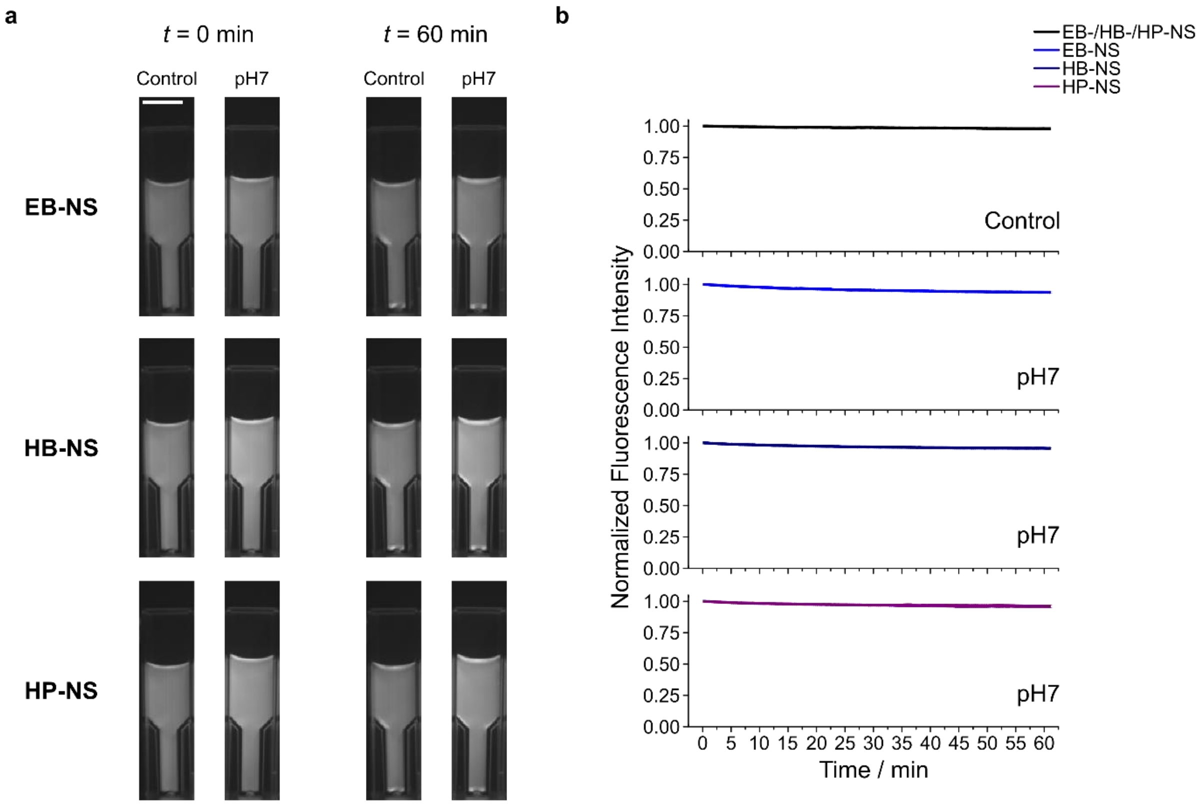

Figure S9. Colloidal stability of EB-NS, HB-NS and HP-NS at neutral pH a Stand-off NIR fluorescence images of NS samples in cuvettes at pH 7. Measurements were taken over 1 h to assess the effect of neutral pH on the stability, *i.e.* the fluorescence of a central region of interest within the cuvette. Control = addition of H_2O (instead of buffer solutions). Scale bar = 1 cm. **b** Corresponding mean fluorescence intensity, normalized to the first frame. All NS displayed a neglectable signal decrease, thus confirming their stability in such conditions. Error bars = standard deviation, $N = 2$ independent samples.

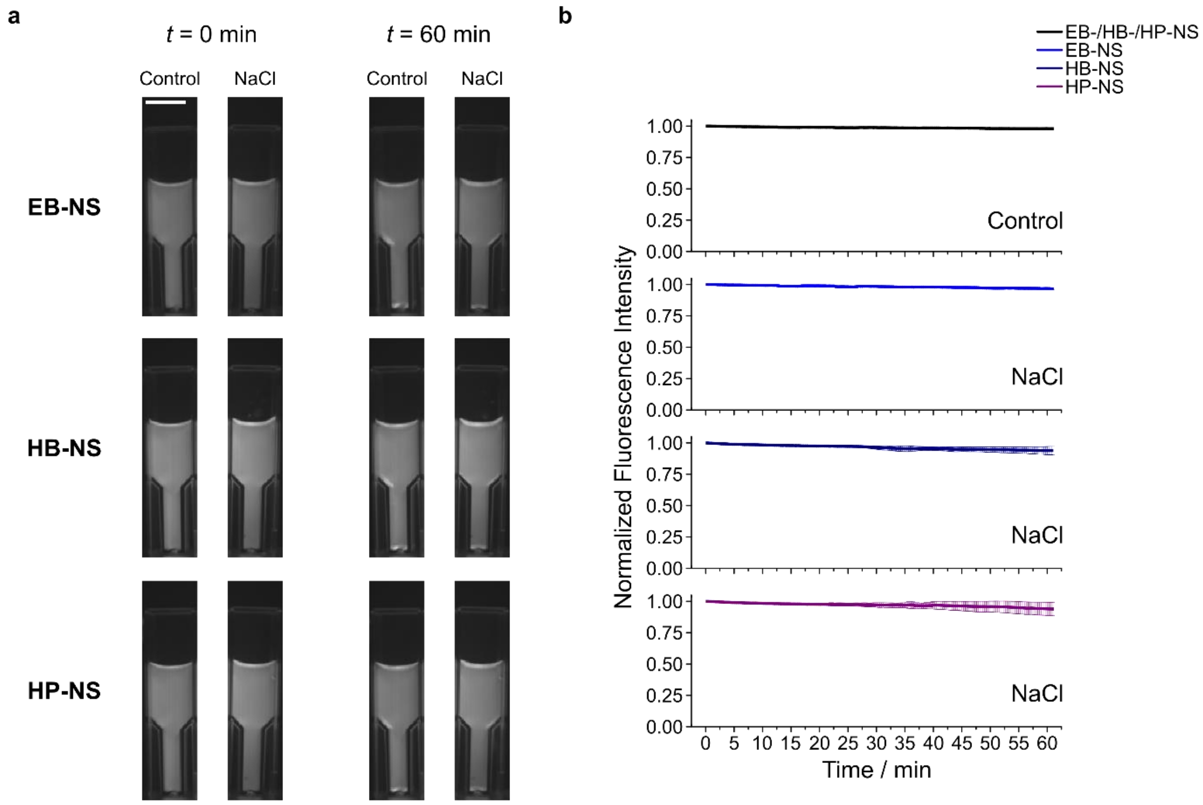

Figure S10. Colloidal stability of EB-NS, HB-NS and HP-NS in ionic environment a Stand-off NIR fluorescence images of NS samples in cuvettes in the presence of sodium chloride (NaCl). Measurements were taken over 1 h to assess the effect of ions (9 g/L NaCl, a typical concentration in blood) on the stability, *i.e.* the fluorescence of a central region of interest within the cuvette. Control = addition of H_2O (instead of NaCl solution). Scale bar = 1 cm. **b** Corresponding mean fluorescence intensity, normalized to the first frame. HB- and HP-NS performed slightly worse than EB-NS. Nevertheless, all NS displayed a signal decrease not lower than 15%, thus proving to be significantly stable in such conditions. Error bars = standard deviation, *N* = 2 independent samples.

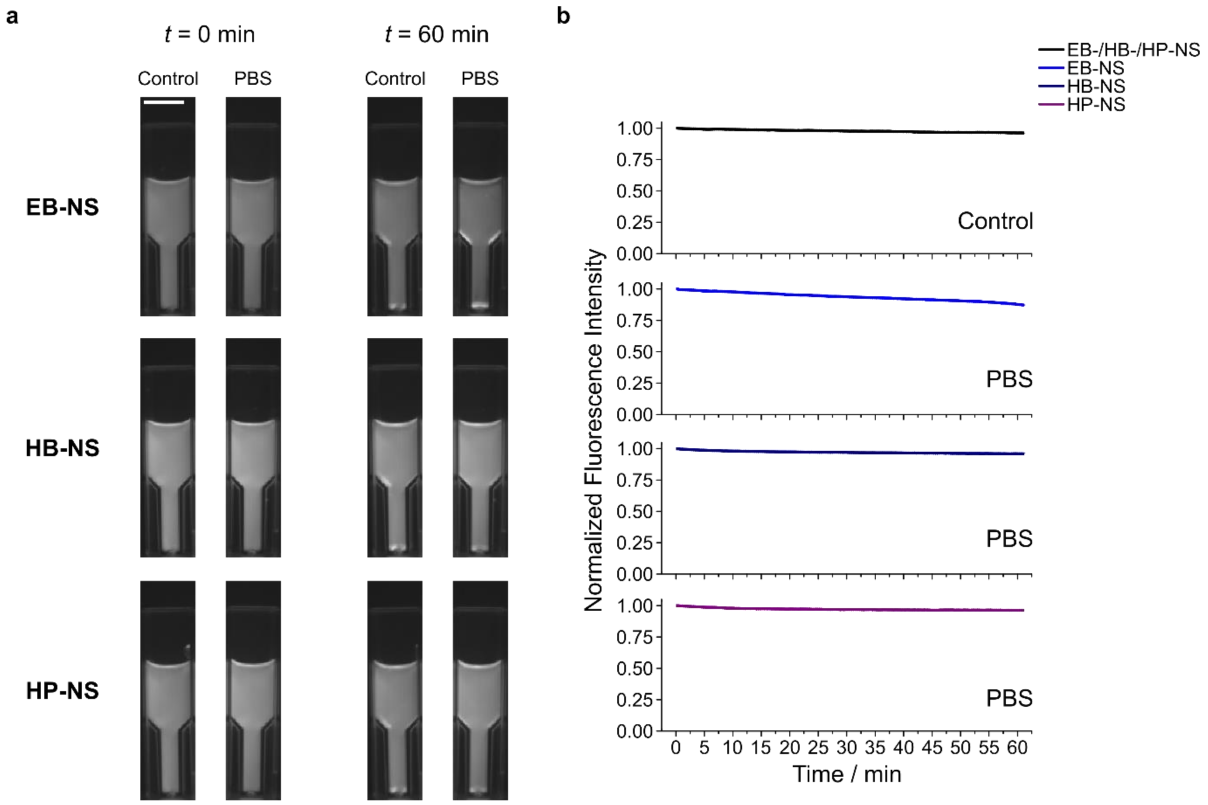

Figure S11. Colloidal stability of EB-NS, HB-NS and HP-NS in buffer solution a Stand-off NIR fluorescence images of NS samples in cuvettes in the presence of buffer (phosphate-buffered saline, PBS). Measurements were taken over 1 h to assess the effect of PBS (1x) on the stability, *i.e.* the fluorescence of a central region of interest within the cuvette. Control = addition of H_2O (instead of buffer). Scale bar = 1 cm. **b** Corresponding mean fluorescence intensity, normalized to the first frame. EB-NS performed slightly worse than HB- and HP-NS. Nevertheless, all NS displayed a signal decrease not lower than 15%, thus proving to be significantly stable in the presence of PBS. Error bars = standard deviation, $N = 2$ independent samples.

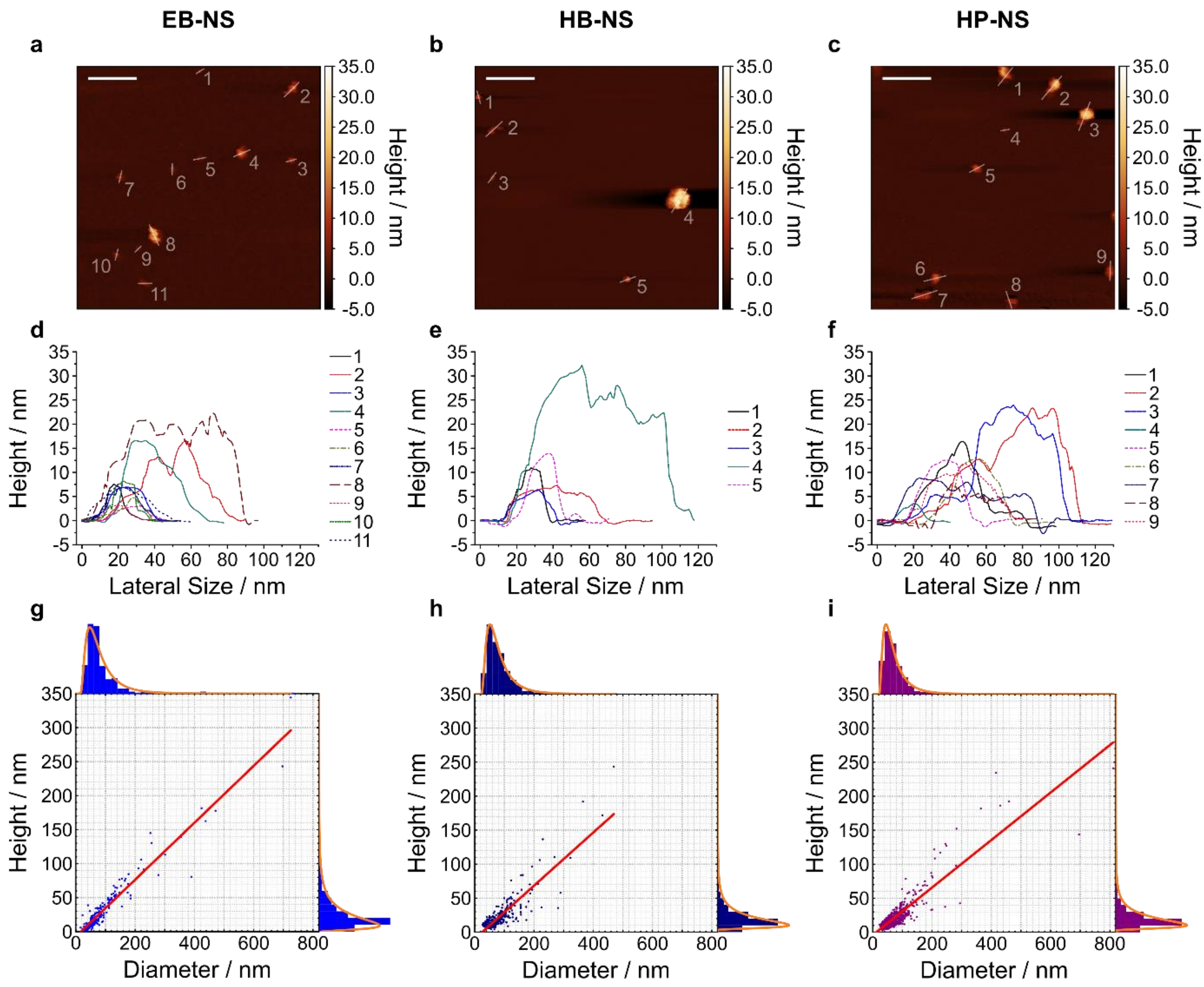

Figure S12. Size and height of exfoliated silicate nanosheets Exfoliated nanosheets were spin-coated on mica and imaged with atomic force microscopy (AFM) in intermittent contact mode. Representative images (**a-c**), height traces (**d-f**) and histograms with log-normal fits (**g-i**) of EB-NS, HB-NS and HP-NS. The results indicate that lateral size scales linearly with height (linear fit = red line in **g-i**). The diameter/height values corresponding to the log-normal distributions' maxima are 47.0 nm / 9.0 nm for EB-NS, 51.5 nm / 10.3 nm for HB-NS, and 44.4 nm / 10.4 nm for HP-NS. R^2 values of the linear fits are 0.924 (EB-NS), 0.755 (HB-NS) and 0.779 (HP-NS). Scale bar = 200 nm. n = 245, 308 and 530 for EB-NS, HB-NS and HP-NS, respectively.

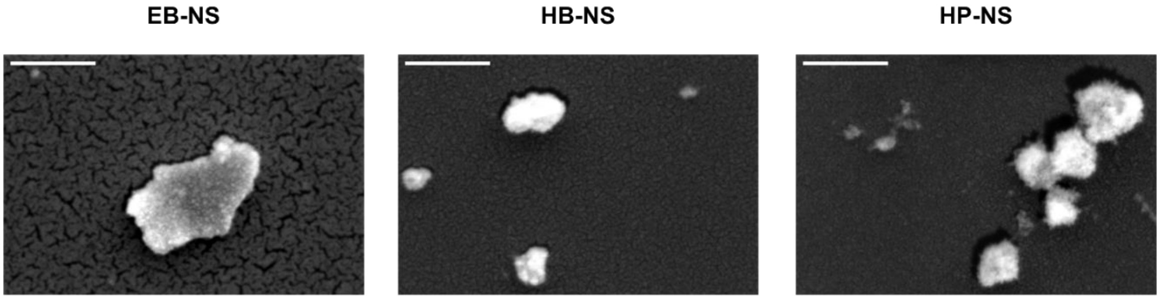

Figure S13. Scanning Electron Microscopy (SEM) images of gold-coated small NS NS with sizes comparable/smaller than the optical resolution limit for optical microscopy ($\lambda \approx 500$ nm, in our case) show a slightly improved contrast after the evaporation of gold on their surfaces. Scale bar = 500 nm.

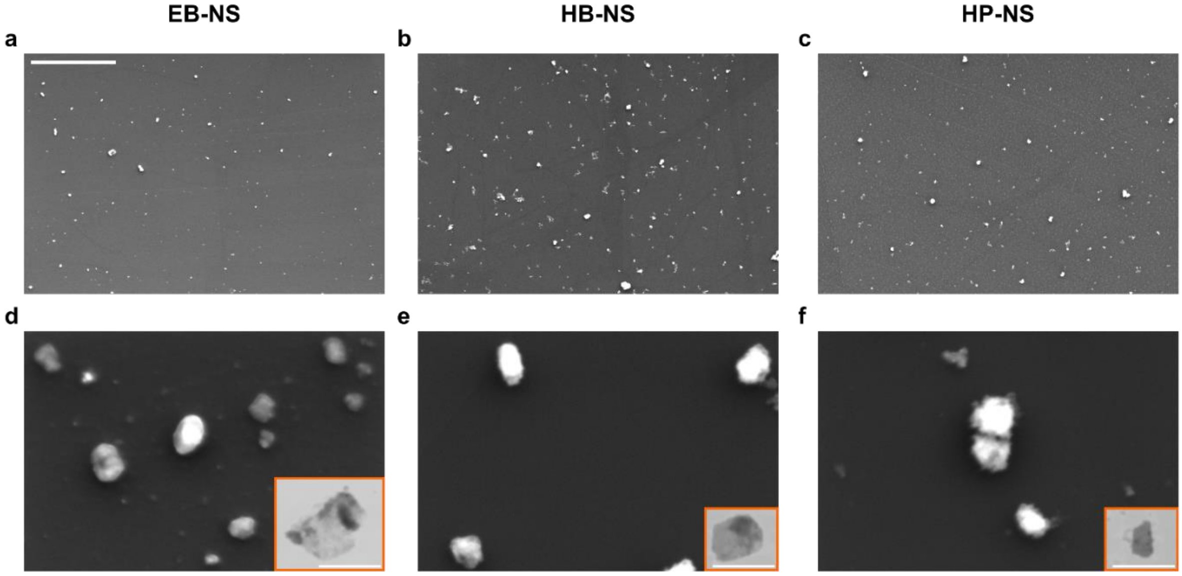

Figure S14. Morphology of nanosheets Scanning electron microscopy (SEM) and scanning transmission electron microscopy (STEM) were used to assess NS morphology**. a-c** EB-NS, HB-NS and HP-NS spin-coated on a graphite substrate. Scale bar = 5 µm. **d-f** Magnified images of the same samples to highlight the presence of particles close to/lower than the resolution limit of optical microscopy. In the lower-right window, clear 2D nanosheet structures could be observed in STEM mode. Scale bar = 500 nm.

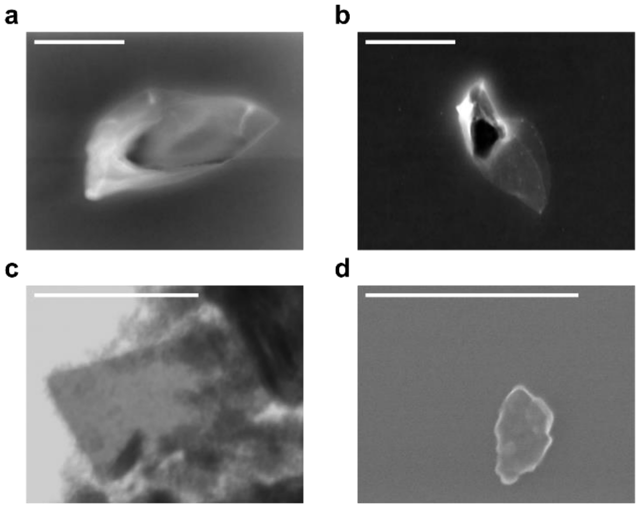

Figure S15. Scanning Transmission Electron Microscopy (STEM) images of small EB-NS a-b EB-NS with sizes slightly larger than Abbe's resolution limit ($\lambda \approx 500$ nm) and displaying the lamellar morphologies typically yielded by fragmentation of thicker EB particles. **c** A thin EB-NS with lateral sizes in the range of the optical resolution limit. **d** Example of an EB-NS of diameter below 500 nm. Scale bar = 500 nm.

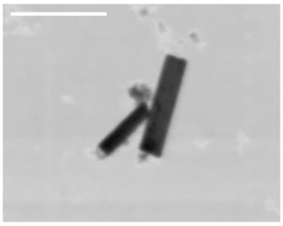

Figure S16. Scanning Electron Microscopy (STEM) images of regular 2D structures in HP-NS Regular 2D structures of HP-NS were noticed, both in SEM and STEM mode. Scale bar = 500 nm.

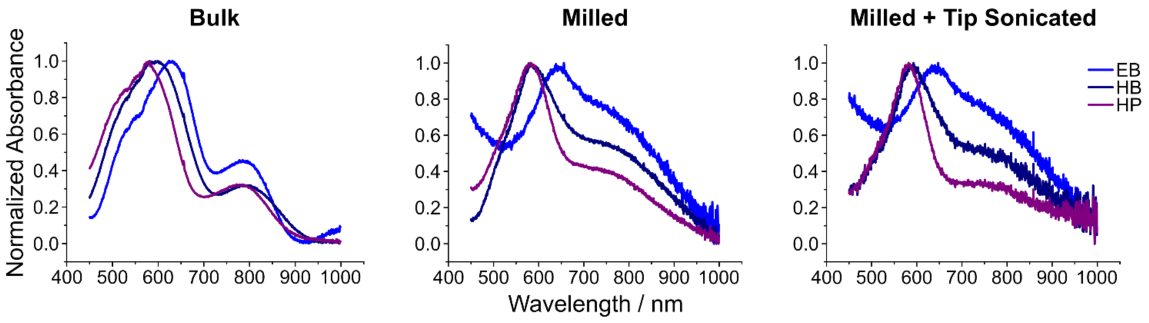

Figure S17. Absorption spectra of bulk, ball milled and fully processed nanosheets of EB, HB and HP Absorbance spectra show impact of processing. First of all, HB is the only powder to show a blue-shift after milling. The main peak position of the other samples is not altered. However, all milled powders lose some features on both the left and right shoulder of the main peak. Features on the right-handed side of the main peak of milled and milled + tip sonicated samples can still be noticed, even if they are not as pronounced as in the bulk counterpart.

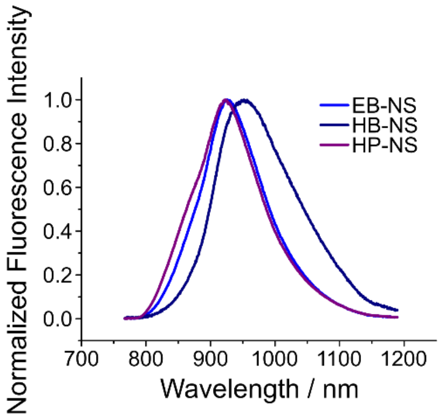

Figure S18. 1D Fluorescence Spectra of NS Normalized NIR fluorescence spectra of EB-NS, HB-NS and HP-NS are plotted. λ_{exc} = 561 nm, laser power = 100 mW and exposure time = 1 s. $\lambda_{emi,\ EB\text{-}NS} \approx$ 927.3 nm (FWHM ≈ 115.3 nm); $\lambda_{emi,\ HB\text{-}NS} \approx$ 953.4 nm (FWHM ≈ 146.6 nm); $\lambda_{emi,\ HP\text{-}NS} \approx$ 923.9 nm (FWHM ≈ 123.7 nm). A slight discrepancy compared to our 2D dataset is likely the result of data correction for the quantum efficiency of the detector and for the spectral irradiance of the xenon lamp, which was here not performed; additionally, spectra normalization and background subtraction might be responsible for further slight shifts, too.

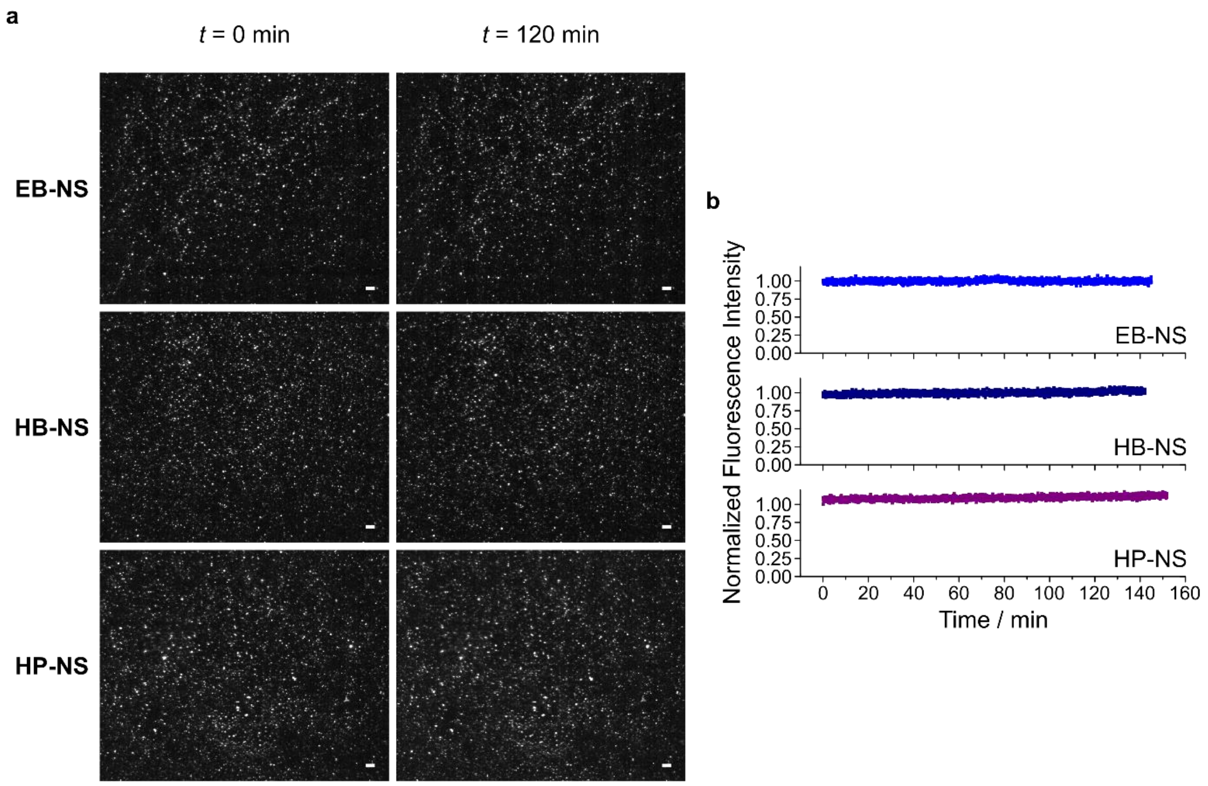

Figure S19. Photobleaching experiments at the microscopic NIR imaging setup a NS were drop-casted and dried on a glass coverslip before a prolonged (t > 2 h) imaging session at an effective output power of ≈ 180 mW. Scale bar = 20 µm. **b** Corresponding mean fluorescence intensity, normalized to the first frame, showing absence of bleaching for all NS. $N = 1$ independent sample.

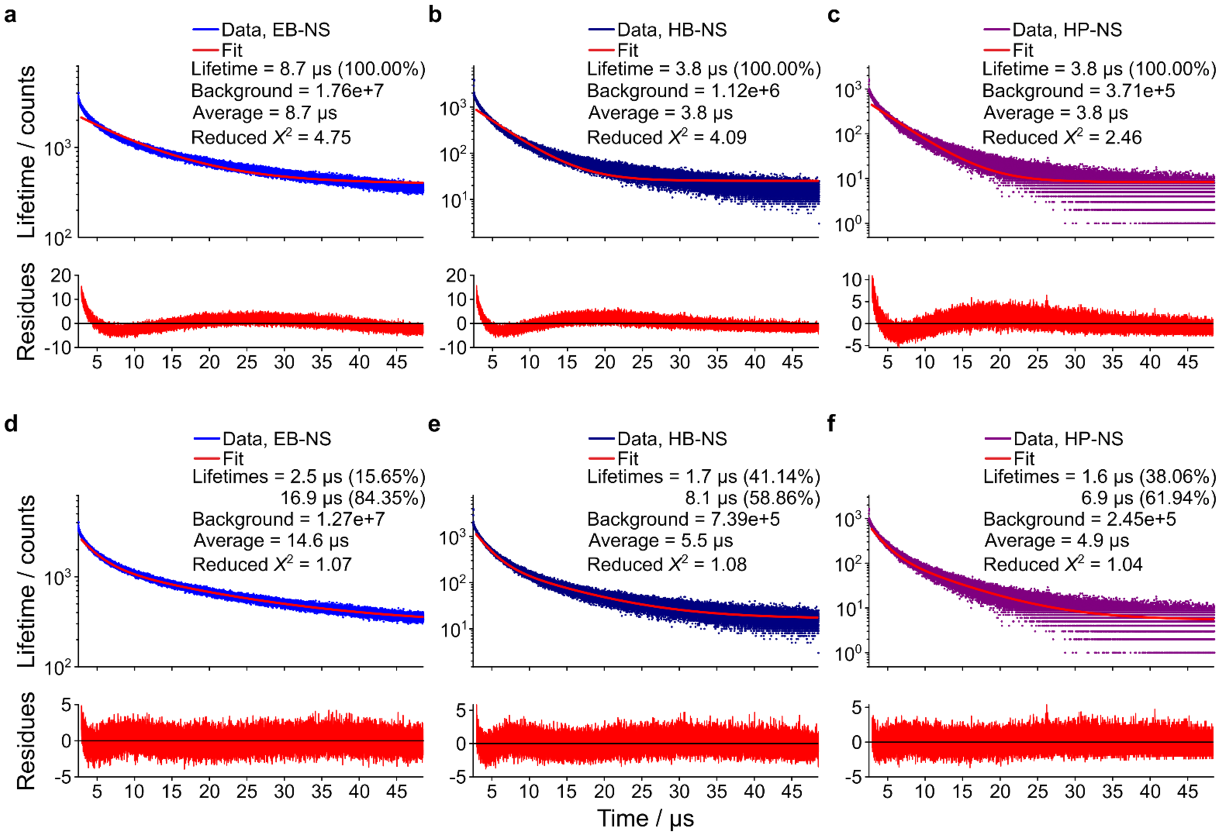

Figure S20. Characterization of the NS fluorescence lifetimes using confocal time-correlated single-photon counting (TCSPC)-based technique Exemplary TCSPC curves of EB-NS, HB-NS and HP-NS with the correspondent single- (**a-c**) and double-exponential (**d-f**) fits. The fit outputs are given on the plots. The fit residues are shown below the fit to guide the eye.

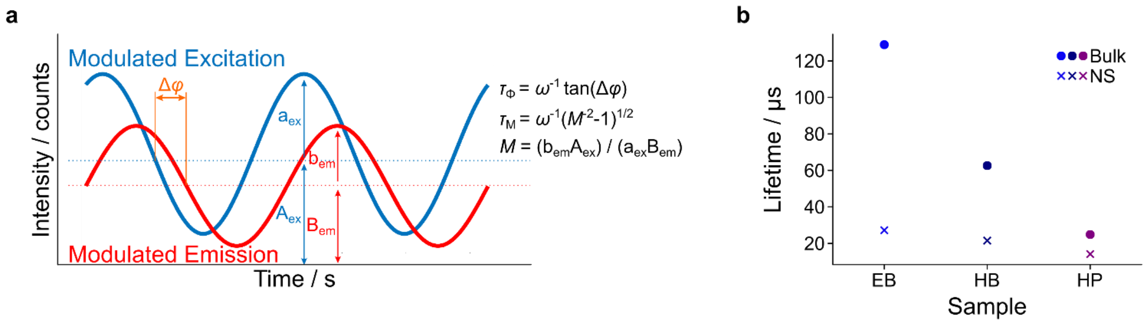

Figure S21. Characterization of fluorescence lifetimes of NS in the frequency domain **a** Schematic describing fluorescence lifetime measurements in the frequency domain. τ_ϕ and τ_M represent the lifetime values obtainable from the phase (ϕ) and the modulation (M), respectively. **b** Frequency domain measurements of bulk and corresponding NS samples of EB, HB and HP performed with a FireSting oxygen sensor device. A decrease in τ is observable for all exfoliated silicates, especially EB-NS. τ_{EB} = 128.89 ± 0.45 µs, $\tau_{EB\text{-}NS}$ = 27.22 ± 0.05 µs, τ_{HB} = 62.60 ± 0.21 µs, $\tau_{HB\text{-}NS}$ = 21.55 ± 0.05 µs, τ_{HP} = 24.85 ± 0.14 µs, $\tau_{HP\text{-}NS}$ = 14.14 ± 0.04 µs. Error bars (not visible by eye) = standard deviation. N = 1 independent sample.

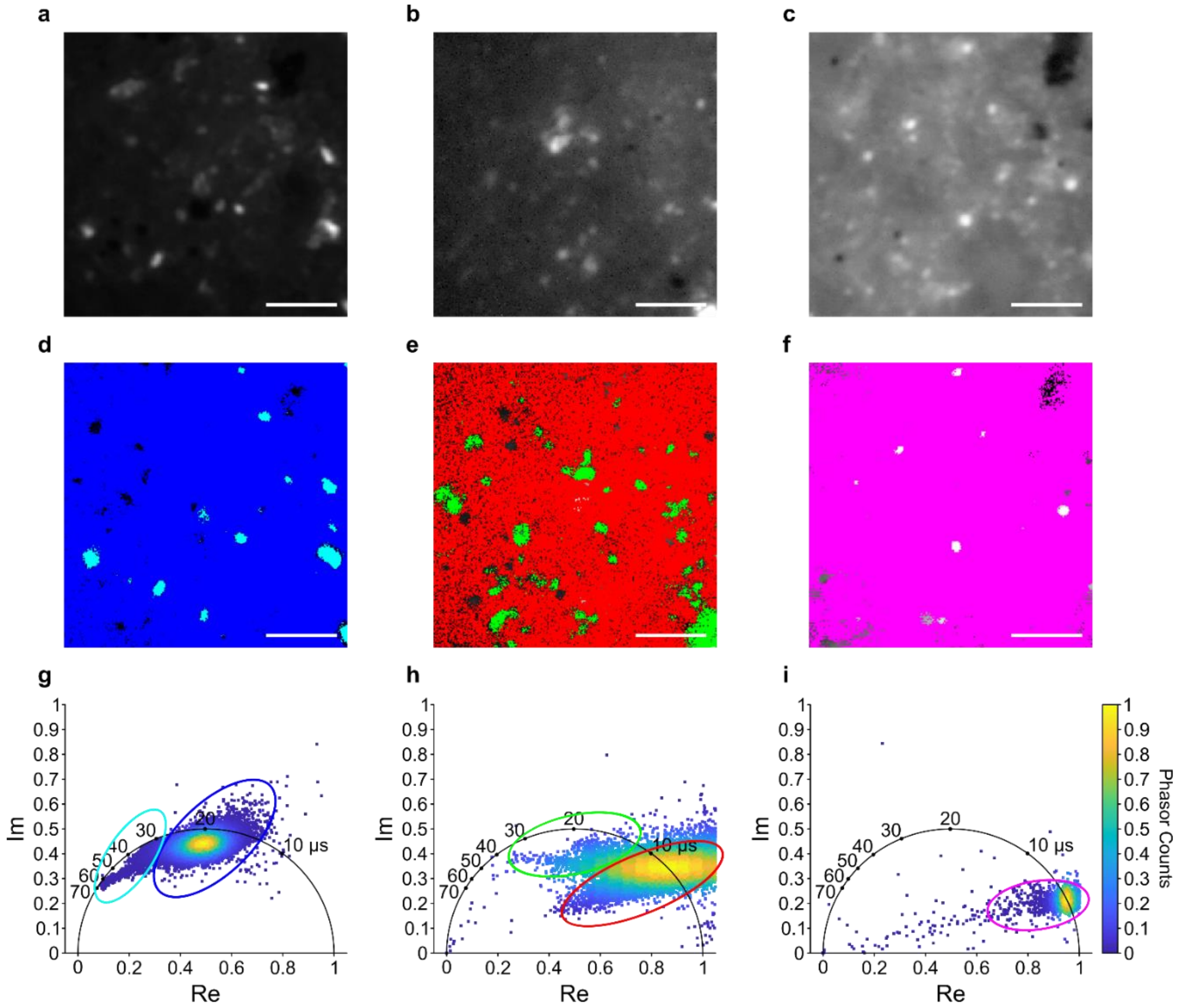

Figure S22. Fluorescence lifetime imaging (FLIM) of the three different NS types using the frequency domain FLIM images of NS drop-casted in a high concentration on top of a glass coverslip: EB-NS (**a**), HB-NS (**b**) and HP-NS (**c**). In **a**, **b** and **c** the intensity images are shown. In **d**, **e** and **f**, the corresponding FLIM images are presented. The colors correspond to a division into the sub-populations displayed in the phasor plots (**g**, **h** and **i**, respectively). Scale bar = 5 µm.

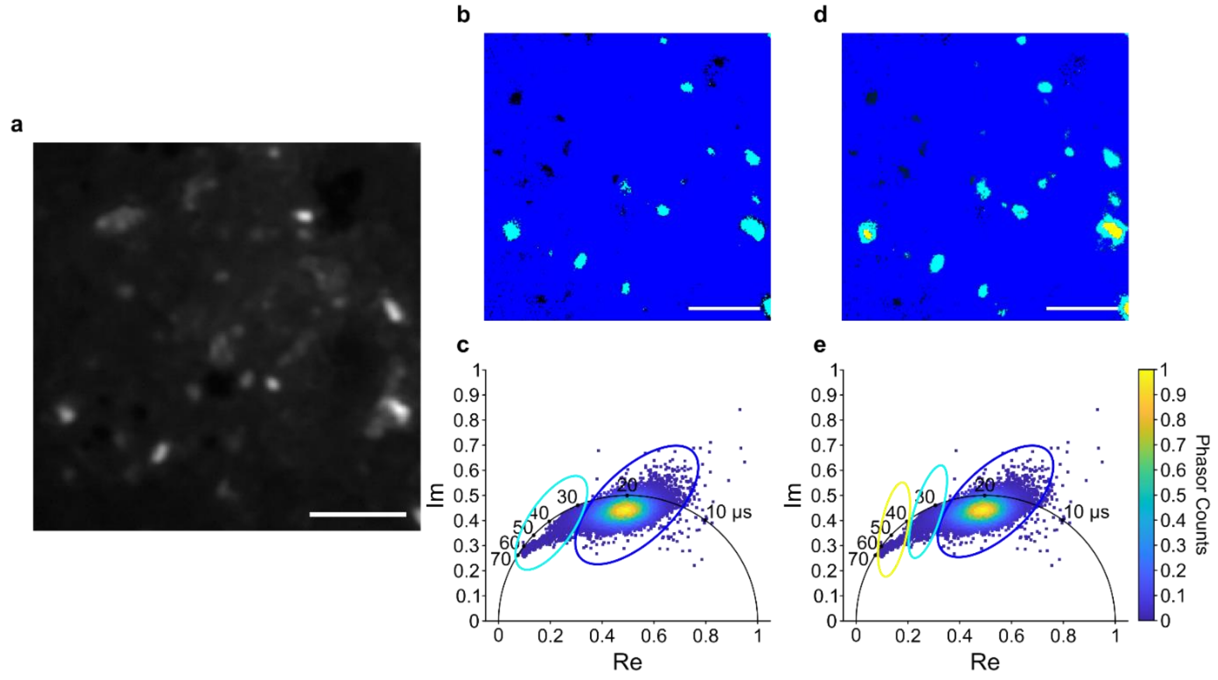

Figure S23. Lifetime-size dependency of EB-NS particles a FLIM image showing the fluorescence intensity of EB-NS drop-casted on top of a glass coverslip. **b** FLIM image of the same sample region in **a**, but with color scheme corresponding to the division of EB-NS into lifetime subpopulations: NS with lifetimes in the range of 15-27 μs appear in dark blue color, the ones in the range of 27-70 μs in light blue color. **c** Phasor plot illustrating the selection of the color scheme in **b**. **d** FLIM image of the same sample region in **a** and **b**, but differently divided into sub-populations: NS with lifetimes in the range of 15-27 μs appear in dark blue color, the ones in the range of 27-40 μs in light blue color and the ones in the range of 40-70 μs in yellow color. **e** Phasor plot illustrating the selection of the color scheme in **d**. Scale bar = 5 μm.

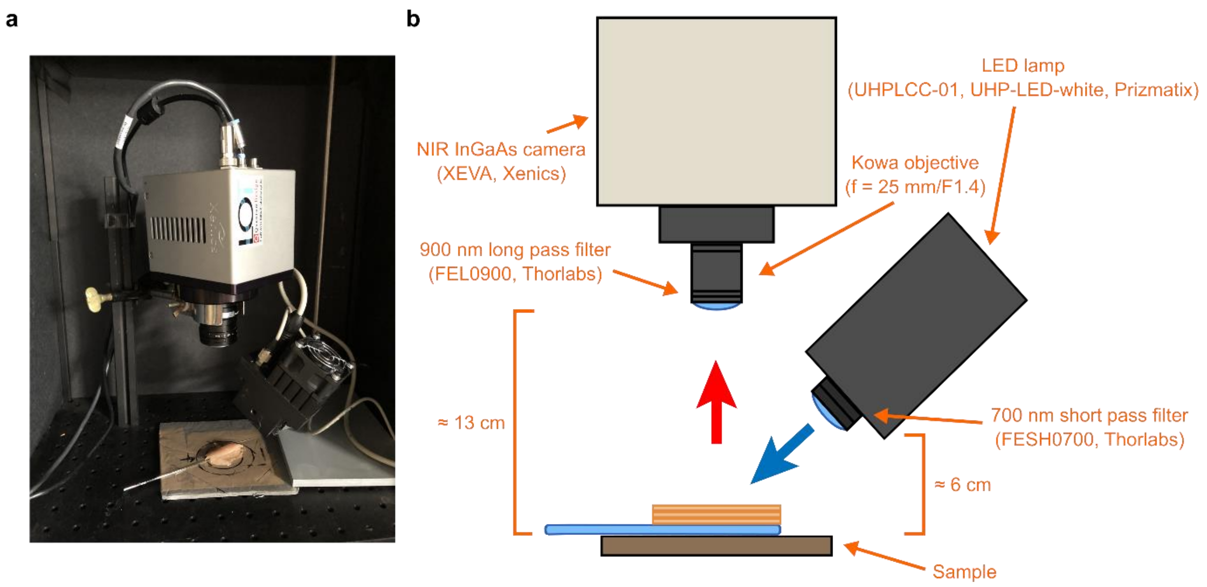

Figure S24. Schematic of home-built stand-off setup a Photo of the home-built stand-off NIR setup, configured for tissue phantom experiments. **b** Corresponding schematic showing the employed devices and filters, as well as the typical working distances.

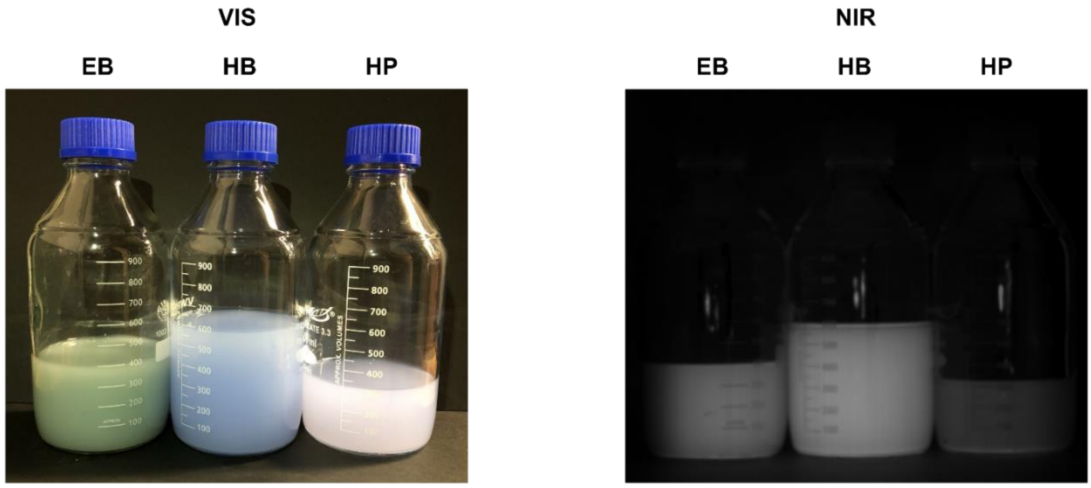

Figure S25. Stand-off imaging of PB milled and centrifuged EB, HB and HP Visible and NIR images acquired at our stand-off setup and showing samples in the exfoliation stage prior to the tip sonication step. The supernatants presented different concentrations, nevertheless they all displayed a clearly detectable fluorescence signal.

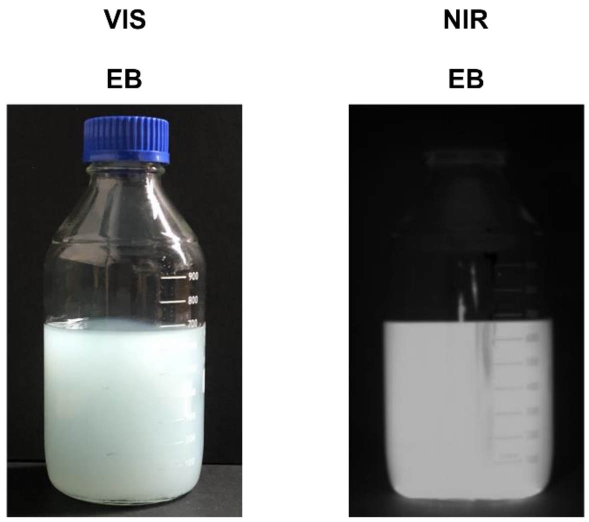

Figure S26. Stand-off imaging of MC milled and centrifuged EB Visible and NIR images acquired at our stand-off setup and showing a MC milled and centrifuged EB sample. The supernatant presented different concentrations compared to other (*e.g.* PB milled) batches, nevertheless the fluorescence signal was clearly detectable.

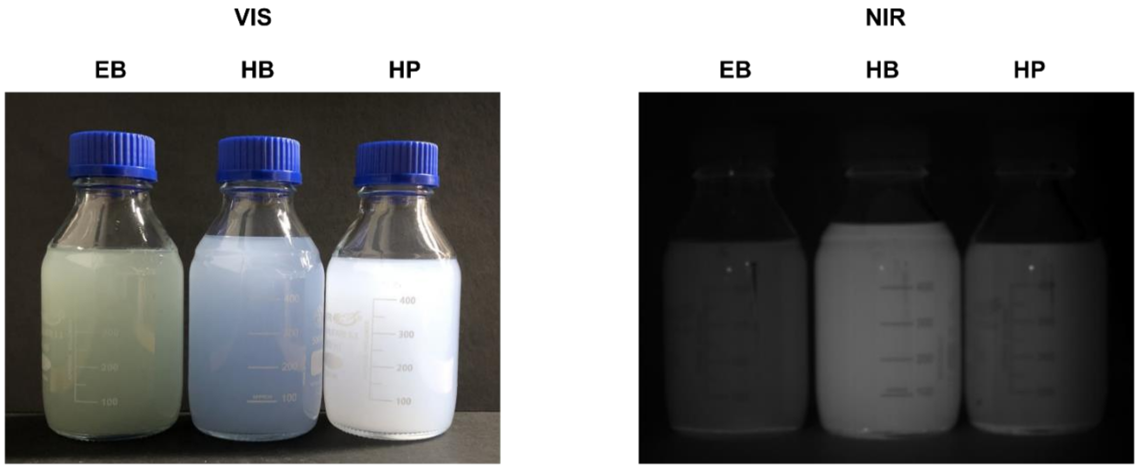

Figure S27. Stand-off imaging of PB+MC milled and centrifuged EB, HB and HP Visible and NIR images acquired at our stand-off setup and showing silicate dispersions obtained *via* a mixed milling approach followed by a centrifugation step. The supernatants presented different concentrations, nevertheless they all displayed a clearly detectable fluorescence signal.

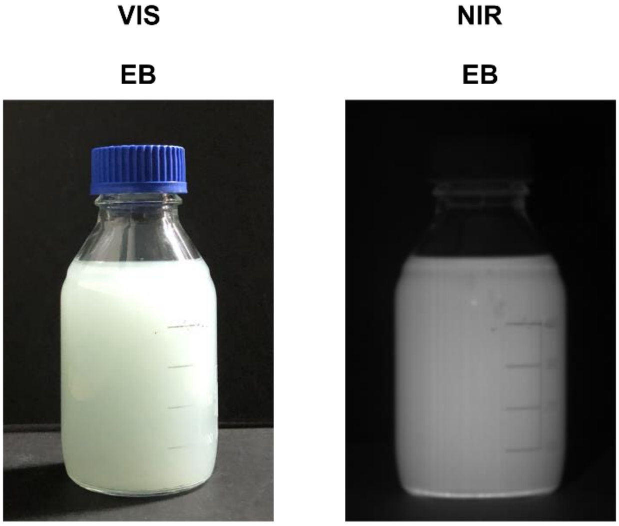

Figure S28. Stand-off imaging of MC+PB milled and centrifuged EB Visible and NIR images acquired at our stand-off setup and showing an EB dispersion obtained *via* a mixed milling approach followed by a centrifugation step. The supernatant presented different concentrations compared to other (*e.g.* PB milled) batches, nevertheless the fluorescence signal was clearly detectable.

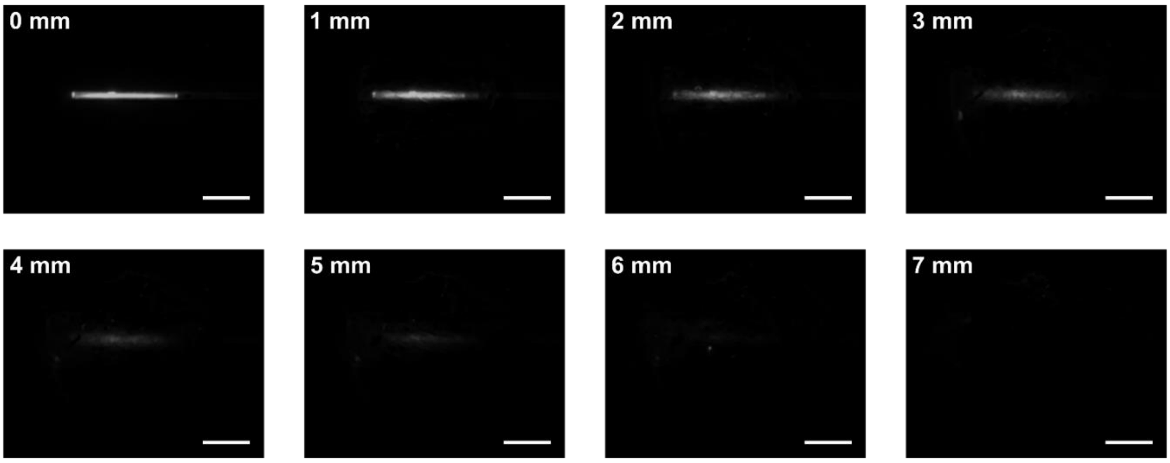

Figure S29. Tissue phantom experiments of EB-NS A concentrated solution of EB-NS (≈ 5 g/L) was sucked into capillary tubes and imaged in a stand-off detection setup. Pictures show NIR fluorescence emitted from the NS under visible excitation (white light source with a 700 nm short-pass filter). The emission signal managed to reach the NIR camera through multiple 1 mm-thick chicken phantom tissues, which were positioned on top of the capillary glass. A background image was subtracted in each image. Scale bar = 10 mm. N = 1 sample.

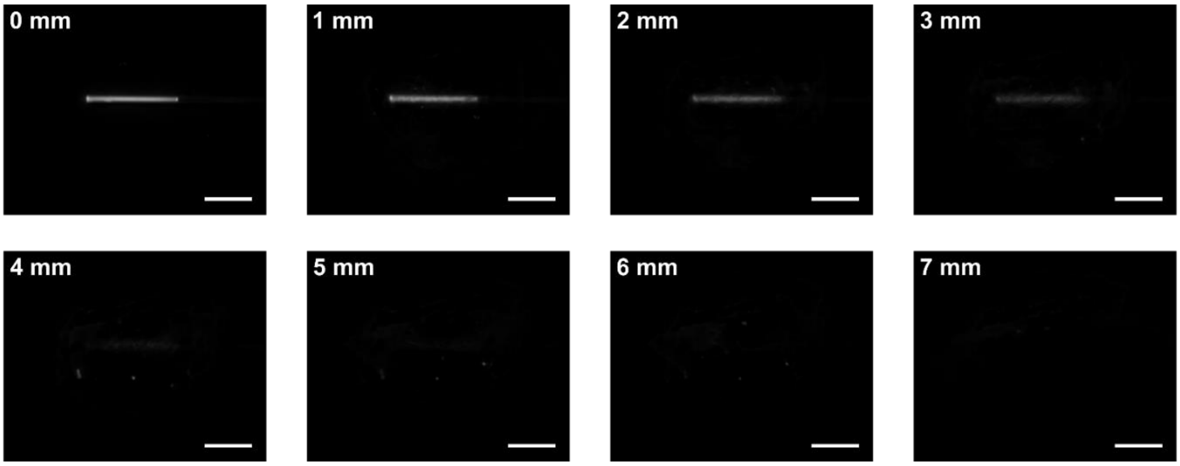

Figure S30. Tissue phantom experiments of HB-NS A concentrated solution of HB-NS (≈ 5 g/L) was sucked into capillary tubes and imaged in a stand-off detection setup. Pictures show NIR fluorescence emitted from the NS under visible excitation (white light source with a 700 nm short-pass filter). The emission signal managed to reach the NIR camera through multiple 1 mm-thick chicken phantom tissues, which were positioned on top of the capillary glass. A background image was subtracted in each image. Scale bar = 10 mm. *N* = 1 sample.

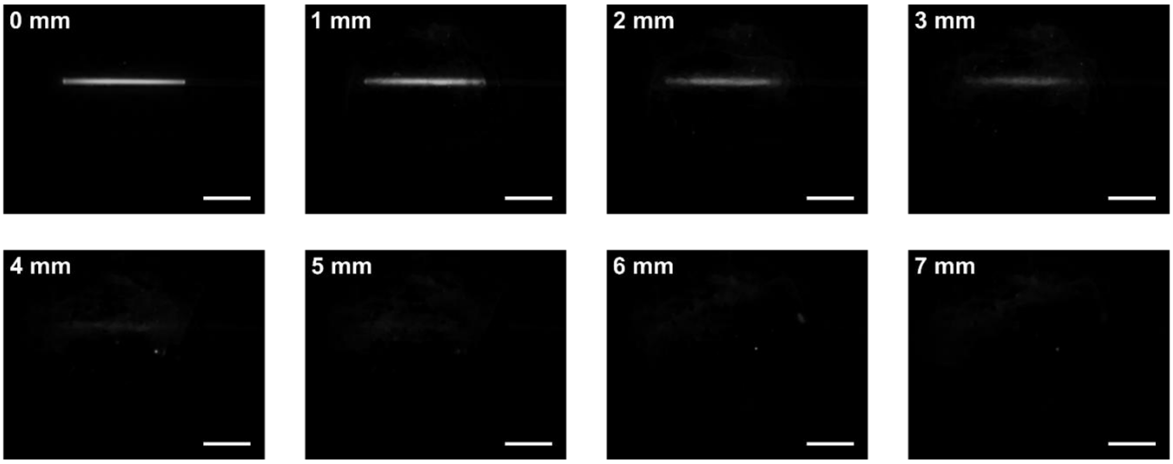

Figure S31. Tissue phantom experiments of HP-NS A concentrated solution of HP-NS (≈ 5 g/L) was sucked into capillary tubes and imaged in a stand-off detection setup. Pictures show NIR fluorescence emitted from the NS under visible excitation (white light source with a 700 nm short-pass filter). The emission signal managed to reach the NIR camera through multiple 1 mm-thick chicken phantom tissues, which were positioned on top of the capillary glass. A background image was subtracted in each image. Scale bar = 10 mm. *N* = 1 sample.

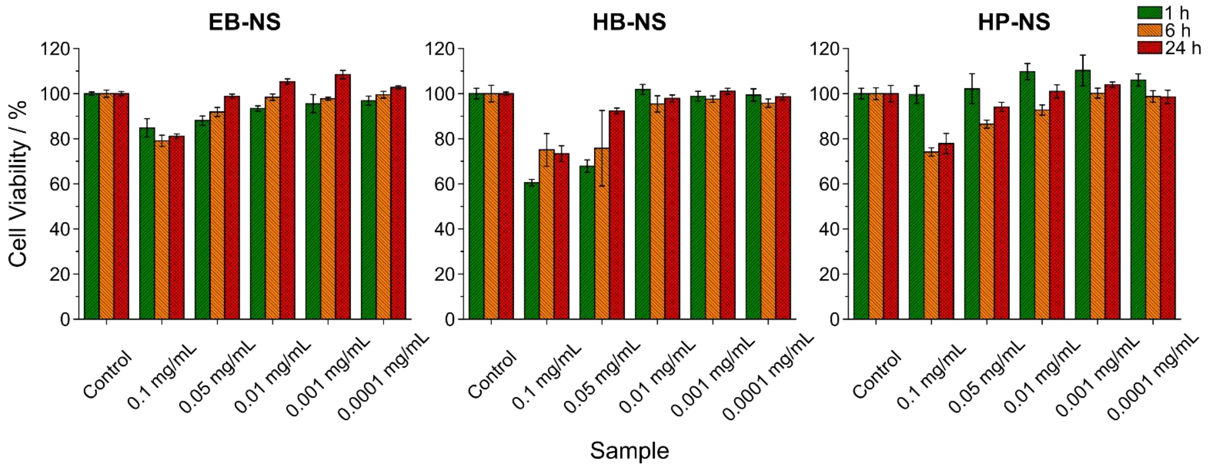

Figure S32. Cytotoxicity tests with cell line 3T3 Different concentrations of EB-NS, HB-NS and HP-NS were dispersed in cell medium and incubated with cell lines of type 3T3 for 1 h, 6 h and 24 h. NS-induced cytotoxicity was assessed *via* a cell proliferation assay whose absorbance at 490 nm is directly proportional to the number of living cells in the culture (expressed in the figure as % cell viability). Only at 0.1 mg/mL (0.05 mg/mL for HB-NS) cell viability decreased slightly. The biologically more relevant lower concentrations showed no effect on viability. Average values calculated from quadruplicates of each sample are plotted. Error bars = standard deviation.

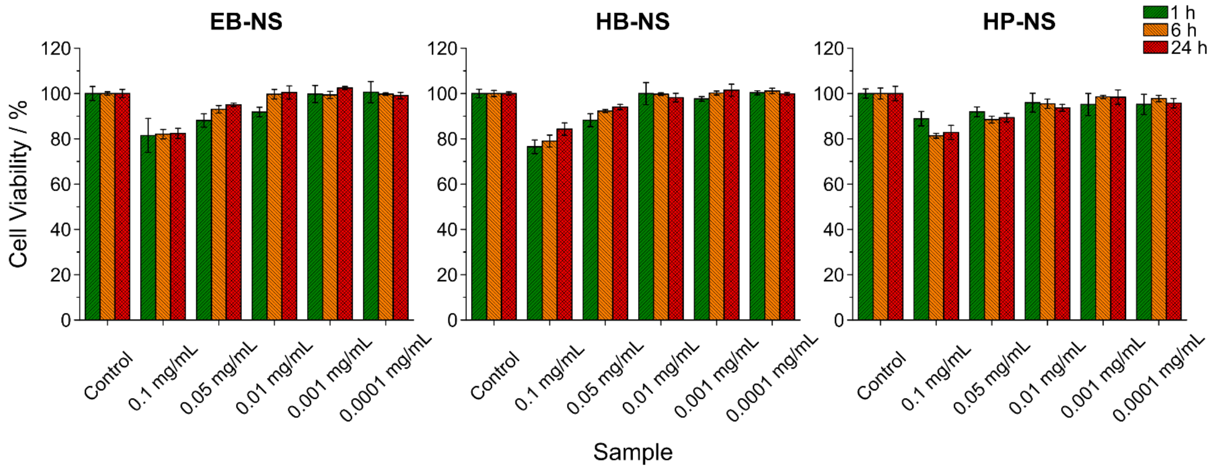

Figure S33. Cytotoxicity tests with cell line A549 Different concentrations of EB-NS, HB-NS and HP-NS were dispersed in cell medium and incubated with cell lines of type A549 for 1 h, 6 h and 24 h. NS-induced cytotoxicity was assessed *via* a cell proliferation assay whose absorbance at 490 nm is directly proportional to the number of living cells in the culture (expressed in the figure as % cell viability). Only at 0.1 mg/mL cell viability decreased slightly. The biologically more relevant lower concentrations showed no effect on viability. Average values calculated from quadruplicates of each sample are plotted. Error bars = standard deviation.

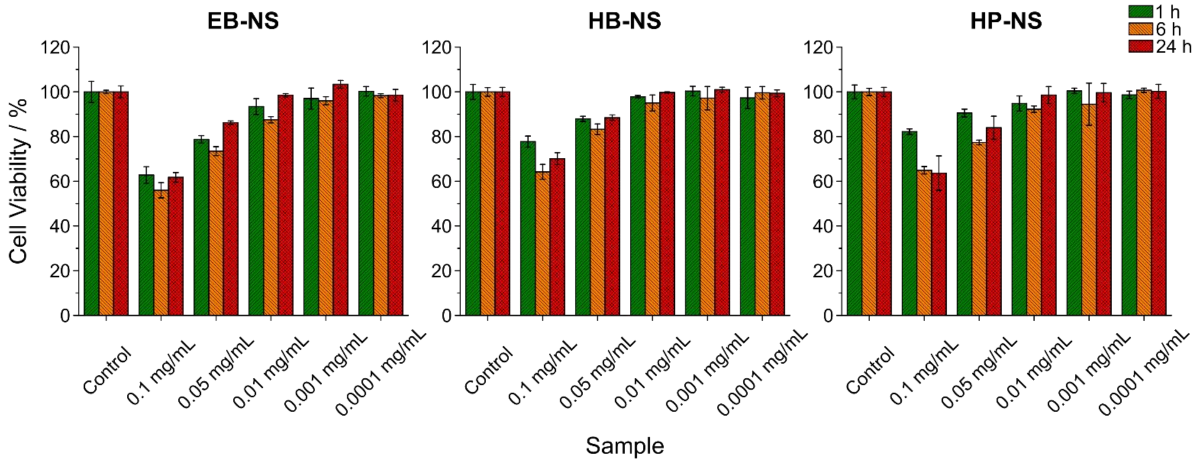

Figure S34. Cytotoxicity tests with cell line MDCK-II Different concentrations of EB-NS, HB-NS and HP-NS were dispersed in cell medium and incubated with cell lines of type MDCK-II for 1 h, 6 h and 24 h. NS-induced cytotoxicity was assessed *via* a cell proliferation assay whose absorbance at 490 nm is directly proportional to the number of living cells in the culture (expressed in the figure as % cell viability). Only at 0.1 mg/mL cell viability decreased slightly. The biologically more relevant lower concentrations showed no effect on viability. Average values calculated from quadruplicates of each sample are plotted. Error bars = standard deviation.

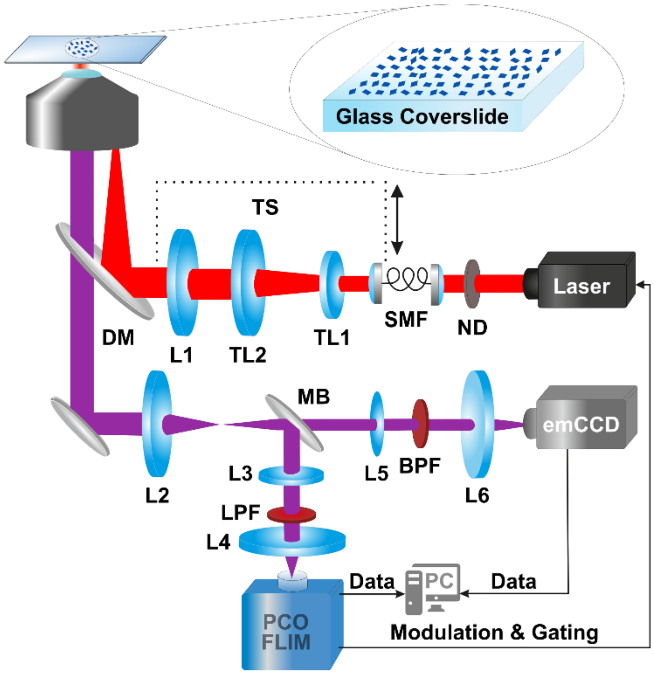

Figure S35. Microscopic fluorescence lifetime imaging (FLIM) on a home-built setup Schematic of the custom-built optical setup for FLIM imaging of NS samples using a frequency domain-based lifetime camera.

Table S1. Comparison of fluorescence lifetime values of EB-NS, HB-NS and HP-NS measured using different experimental techniques: confocal TCSPC, microscopic PCO.FLIM lifetime imaging and oxygen sensor For TCSPC measurements, the mean value and standard deviation were calculated from at least three separate measurements performed on the same samples (N = 3-4 measurements). For FLIM imaging, the lifetime values of each pixel of the acquired image were histogrammed and fitted with the single Gaussian fit; the mean lifetime and standard deviation were taken from the fit (N = 1 measurement). For the modulated LED dataset, the error bar simply corresponds to the standard deviation of the acquisition (N = 1 measurement).

Sample	τ_{TCSPC} [μs]	$\tau_{FLIM\ imaging}$ [μs]	$\tau_{modulated\ LED}$ [μs]
EB-NS	16.50 ± 0.25	17.6 ± 2.4	27.22 ± 0.05
HB-NS	8.25 ± 0.15	7.8 ± 1.5	21.55 ± 0.05
HP-NS	6.91 ± 0.06	4.5 ± 0.7	14.14 ± 0.04

SUPPLEMENTARY EXPERIMENTAL SECTION

Absorption (Reflection) Spectra of Egyptian Blue (EB), Han Blue (HB) and Han Purple (HP) Bulk and Exfoliated Powders: Bulk powders were analyzed with no prior processing. Aliquots of the milled supernatants were positioned on a watch glass under the lab hood and left to dry in air over several days. Aliquots of the fully exfoliated NS (milled and tip sonicated supernatants) were instead freeze-dried for a couple of days, due to the lower concentrations of this batch of samples. To record absorption spectra, an AvaSpec-UV/Vis/NIR two channel broad band spectrograph (Avantes) equipped with a balanced deuterium-halogen lamp (AVALIGHT-DH-S-BAL) was employed. For our purposes, only the signal recorded in the UV/Vis channel is relevant, therefore the NIR one is not shown; more precisely, the settings of the 2048L UV/Vis spectrometer included a 25 µm slit and a 300 lines/mm grid. Data acquisition and analysis were performed with the Avasoft-Full and Origin Pro 8.1 software, respectively.

Planetary Ball (PB), McCrone (MC) and Mixed Milling Approaches: EB, HB and HP powders (respectively < 120 µm, < 40 µm and < 40 µm qualities) were purchased from Kremer Pigmente GmbH & Co. KG. The standard milling procedure for the first phase of exfoliation was planetary ball (PB) milling, which was performed as follows. 3 g of powder were introduced into a 20 mL agate beaker together with 5 mm agate balls. Deionized water was added until a slurry consistency was reached. The so-prepared agate beaker was then placed in a planetary ball mill (PB, Pulverisette 7 Premium Line, Fritsch, Germany), which was run at 900 r.p.m. for 1 h (3 cycles of 20 min each, 5 min pause). Aliquots of the resulting milled slurry were removed from the beaker and their grain size distributions were measured by means of a laser diffraction particle sizer (LDPS, model LS13320, Beckman&Coulter). For each measurement, three runs were performed, PIDS was used, and an optical model R.I. 1.6/1 was

applied. To visualize the grain size distribution, the number of particles within an individual grain size class was plotted as percentage of all particles against the central diameter of the class. In this way, the very small number of bigger particles present in the system does not dominate the distribution curve, as it would instead happen if volume percentage were displayed. Following the PB milling step, an aliquot was poured into a Nalgene® centrifuge tube (Thermo Fisher Scientific) and water was added until an overall volume of 150 mL (dilution factor ≈ 3) was reached. Then, a first centrifugation step (Heraeus Multifuge X3R, Thermo Fisher Scientific) was performed: T = 20°C, 800 r.p.m. (150 × g), 9 min 41 s, 5 s acceleration ramp, 5 s deceleration ramp, 5 cycles. These parameters were calculated from the Stokes Equation (corrected for centrifugation[1]) in order to remove particles of diameter $d > 1$ μm:

$$t = \frac{18 \times \eta}{(\rho_k - \rho_w) \times 4\pi^2 \times f^2 \times d^2} \times \ln \frac{r}{r_0} . \quad \text{(S1)}$$

In Equation (S1), t is the settling time [s], η the temperature-dependent dynamic viscosity of water [kg m^{-1} s^{-1}], ρ_k the grain density [kg m^{-3}], ρ_w the temperature-dependent water density [kg m^{-3}], f the r.p.m. [s^{-1}], d the previously mentioned grain (equivalent) diameter [m], r the distance between the rotor's fulcrum and the sediment's height [cm], and r_0 the distance between the rotor's fulcrum and the suspension's surface [cm] (the last two parameters are dependent on the geometry of the employed centrifuge). At the end of each cycle, the supernatant was decanted and new water was added up to the initial volume; finally, the so-obtained supernatant dispersion was collected in a glass jar and stored at room temperature. New LDPS measurements were performed to check the efficiency of the described centrifugation step in terms of the resulting size distribution. Aliquots of each supernatant of each of the three milled pigments were taken, dried and weighed, yielding the following concentrations: EB ≈ 2.2 g/L, HB ≈ 1.4 g/L and HP ≈ 0.6 g/L. While EB proved to be stable for up to ≈ 2 days already at this stage, milled HB and HP particles settled after a few hours.

To understand whether better results (*i.e.* smaller grain size and higher monodispersity) could be obtained *via* techniques other than PB milling, also McCrone (MC) and a mixed milling approach were tested with some of the mentioned powders. With regards to the former, 2 g of bulk EB were added into MC beakers together with 48 agate cylinders and ≈ 7 mL of demineralized water; 2 consecutive milling cycles of 30 min each were performed (XRD-Mill McCrone, Retsch), then the slurry was washed out of the beaker to yield ≈ 45 mL of total slurry volume.

Concerning the mixed approaches, instead, the starting material was represented by the settled parts of PB and MC centrifugations (*i.e.* the sediment, which had been collected and dispersed in demineralized water): according to the order these millings were executed in, the samples were named “PB+MC” or “MC+PB”. The former ones were prepared by loading a total volume of 10 mL of sediment slurries into the MC mill for 30 min: after washing steps, a final volume of ≈ 45 mL was collected. For “MC+PB” samples, instead, MC sediment slurries were first centrifuged according to the following settings: T = 20°C, 3000 r.p.m. (2103 × g), 5 min, 5 s acceleration ramp, 5 s deceleration ramp, 1 cycle. Next, the so-obtained volume of sediment (≈ 8 mL) was placed in PB beakers and milled as described above: after washing steps, a final volume of ≈ 45 mL could be collected.

For MC and mixed milling techniques alike, samples were diluted and centrifuged to produce the supernatant slurries, analogously to how described for the (reference) PB approach. For all exfoliation routes, size distribution measurements of “whole” and “centrifuged” samples were carried out by means of LDPS before and after the centrifugation step.

Scanning Electron Microscopy (SEM) and Scanning Electron Transmission Microscopy (STEM): For SEM imaging (Quattro S SEM, Thermo Fisher Scientific), highly-oriented pyrolytic graphite (HOPG, grade ZYB, Bruker) were used as substrates. HOPGs were plasma-

treated (Zepto Diener Electronic GmbH +Co. KG, 1 min of O_2 supply, 1 min of plasma process) in order to clean their surfaces and increase their hydrophilicity. Next, a typical NS sample was vortexed and bath sonicated for 10 min. 10 µL of undiluted sample were spin-coated with the same parameters employed for AFM measurements. For each NS sample, the so-prepared HOPG was either imaged at the SEM as it is, or a ≈ 4 nm-thick gold layer was evaporated (Baltec MED-020, Baltec) onto it to decrease surface charging and, thus, increase imaging contrast. The HOPG was then placed into the SEM chamber and imaged in the following conditions: high vacuum mode, voltage = 5.00 kV, spot size = 3.0, working distance = 10.0 mm, Everhart-Thornley detector (ETD) for secondary electrons, Circular Backscatter Detector (CBS) for backscattered electrons.

STEM measurements were carried out on the same device. Typically, undiluted 5 µL of previously vortexed and bath sonicated NS were deposited and dried onto formvar-coated copper grids, stabilized with evaporated carbon film (FCF300-CU, Electron Microscopy Sciences). The parameters are the same as for SEM imaging, except for the employed detector (STEM3+).

Photobleaching Experiments at the Near-Infrared (NIR) Imaging Microscopic Setup: The imaging setup consists of a 561 nm laser (Cobolt JiveTM 561 nm), an Olympus IX53 microscope equipped with a 20x (MPlanFL N 20x/0.45, Olympus) objective, and a NIR camera (Cheetah TE1, Xenics). Along the light path leading from the microscope to the camera, a dichroic mirror (VIS/NIR, HC BS R785 lambda/5 PV, F38-785S, AHF) and a 900 nm long-pass filter (FELH0900, Thorlabs) are installed. To investigate whether NS can be bleached, a long imaging session with continuous excitation was performed on 10 µL of NS sample, previously drop-casted and dried on a #1 glass coverslip. Acquisition settings were the following: laser set power = 500 mW, measured power out of the objective ≈ 180 mW,

acquisition time ≈ 2 h, exposure time = 0.5 s, frame rate = 0.3 fps. Data acquisition was controlled by the Xenics software (v. 2.6), whereas data analysis was carried out in ImageJ (v. 1.52a) and on Origin Pro 8.1.

NIR Imaging at Stand-Off Detection Setup: Our NIR custom-made stand-off detection setup[2] consists of a NIR InGaAs camera (XEVA, Xenics), a Kowa objective (f = 25 mm/F1.4), and a white light source (UHPLCC-01, UHP-LED-white, Prizmatix). The latter is equipped with a 700 nm short pass filter (FESH0700, Thorlabs) for excitation. Furthermore, a 900 nm long pass filter (FEL0900, Thorlabs) is mounted on the camera.

For imaging of EB, HB and HP water dispersions in glass vials, an exposure time of 3 s at maximum lamp intensity was employed.

Colloidal stability experiments were performed in cuvettes on sample volumes of ≈ 2 mL. EB-NS, HB-NS and HP-NS had a starting pH value in the range of ≈ 8-9. Prior to imaging, NS were bath sonicated for 5 min. Then, buffer solutions of pH 4 and 7 were added to some NS cuvettes in the volumes necessary to reach pH 5 and pH 7, respectively. To study the impact of ions on the colloidal stability of the NS, 200 µL of a solution of NaCl (9 g/L, a typical blood concentration) were added into other cuvettes. Finally, control samples consisted in the addition of 200 mL of H_2O. Imaging was started shortly after the introduction of the mentioned aliquots in the cuvettes. Acquisition settings were: exposure time = 2 s, frame rate = 0.067 fps, LED excitation power = 50%, acquisition time ≈ 60 min, LED-cuvette distance ≈ 18 cm, camera-cuvette distance ≈ 20 cm. The acquired images were processed as is, *i.e.* without background subtraction. To assess the fluorescence intensity over time, the mean signal intensity in a central region of interest of each cuvette was measured (and normalized to the starting frame).

Concerning sample preparation for tissue phantom experiments, 1 mL of NS batch was concentrated (Concentrator 5301, Eppendorf) for ≈ 2 h, yielding a volume of ≈ 0.1 mL with

concentration of ≈ 5 g/L. After ≈ 5 min of bath sonication, a capillary tube (ringcaps® 25 µL, Hirschmann Laborgeräte GmbH & Co. KG) was dipped into the NS vial, and NS were thus sucked into the glass tube due to capillary forces. For each class of silicates, a so-prepared capillary tube was positioned onto the setup's stage at ≈ 6 cm and ≈ 13 cm away from the excitation lamp and the detection camera, respectively. Initial reference images were acquired with 0.5 s of exposure time at maximum excitation intensity. Afterwards, ≈ 1 mm-thick chicken slices were step-wise laid on top of the capillary tube, and new images with the same settings were taken after every layer's deposition.

For all acquired datasets (unless explicitly mentioned), background subtraction (*i.e.* subtraction of background reference image) and data analysis of the so-obtained NIR pictures were carried out in ImageJ (v. 1.52a) and Origin Pro 8.1.

Cytotoxicity Tests: Cells were initially seeded in a 96-well plate at 1.2×10^3 cells per well for 24 h in EMEM with Earle's salts with GlutaMAXTM (Gibco) and 10% FCS (BioWest) at 37°C and 7.5% CO_2 (MDCK II), or in DMEM with L-Glutamine (4 mM) and 10% FCS (BioWest) at 37°C and 5% CO_2 (A549 and NIH 3T3). Freeze-dried EB-NS, HB-NS and HP-NS were dissolved at 10 mg/mL in the appropriate media for each cell line with 100 µg/mL Penn-Strep (Biochrom). Different concentrations were subsequently added to the cells and incubated for 1, 6 or 24 h. Viability of the cell samples was determined by MTS-assay using CellTiter 96® AQueous One Solution Cell Proliferation Assay (Promega); for each sample, the absorption at 490-650 nm was measured.

SUPPLEMENTARY REFERENCES

[1] H. Tributh, G. Lagaly, *GIT Fachz. Lab.* **1986**, *30*, 524.

[2] R. Nißler, O. Bader, M. Dohmen, S. G. Walter, C. Noll, G. Selvaggio, U. Groß, S. Kruss, *Nat. Commun.* **2020**, *11*, 1.
